# A genomics approach identifies selective effects of *trans*-resveratrol in cerebral cortex neuron and glia gene expression

**DOI:** 10.1371/journal.pone.0176067

**Published:** 2017-04-25

**Authors:** Gemma Navarro, Eva Martínez-Pinilla, Alejandro Sánchez-Melgar, Raquel Ortiz, Véronique Noé, Mairena Martín, Carlos Ciudad, Rafael Franco

**Affiliations:** 1 CIBERNED. Centro de Investigación Biomédica en Red sobre Enfermedades Neurodegenerativas. Instituto de Salud Carlos III, Madrid, Spain; 2 Institute of Biomedicine of the University of Barcelona (IBUB), Barcelona, Spain; 3 Department of Biochemistry and Molecular Biomedicine, Faculty of Biology, University of Barcelona, Barcelona, Spain; 4 Instituto de Neurociencias del Principado de Asturias (INEUROPA), Departamento de Morfología y Biología Celular, Facultad de Medicina, Universidad de Oviedo, Asturias, Spain; 5 Facultad de Ciencias y Tecnologías Químicas & Facultad de Medicina. Departamento de Química Inorgánica, Orgánica y Bioquímica, Centro Regional de Investigaciones Biomédicas (CRIB), Universidad de Castilla-La Mancha, Ciudad Real, Spain; 6 Department of Biochemistry and Physiology, School of Pharmacy, University of Barcelona, Barcelona, Spain; 7 Institute of Nanotechnology of the University of Barcelona (IN2UB), Barcelona, Spain; Centre National de la Recherche Scientifique, FRANCE

## Abstract

The mode of action of *trans*-resveratrol, a promising lead compound for the development of neuroprotective drugs, is unknown. Data from a functional genomics study were retrieved with the aim to find differentially expressed genes that may be involved in the benefits provided by *trans*-resveratrol. Genes that showed a significantly different expression (p<0.05, cut-off of a two-fold change) in mice fed with a control diet or a control diet containing *trans*-resveratrol were different in cortex, heart and skeletal muscle. In neocortex, we identified 4 up-regulated (*Strap*, *Pkp4*, *Rab2a*, *Cpne3*) and 22 down-regulated (*Actn1*, *Arf3*, *Atp6v01*, *Atp1a3*, *Atp1b2*, *Cacng7*, *Crtc1*, *Dbn1*, *Dnm1*, *Epn1*, *Gfap*, *Hap*, *Mark41*, *Rab5b*, *Nrxn2*, *Ogt*, *Palm*, *Ptprn2*, *Ptprs*, *Syn2*, *Timp2*, *Vamp2*) genes upon *trans*-resveratrol consumption. Network analysis of gene products provided evidence of plakophilin 4 up-regulation as a triggering factor for down-regulation of events related to synaptic vesicle transport and neurotransmitter release via underexpression of dynamin1 and Vamp2 (synaptobrevin 2) as node-gene drivers. Analysis by RT-qPCR of some of the selected genes in a glioma cell line showed that dynamin 1 mRNA was down-regulated even in acute *trans*-resveratrol treatments. Taken all together, these results give insight on the glial-neuronal networks involved in the neuroprotective role of *trans*-resveratrol.

## Introduction

Phytochemicals are in the front line to combat oxidative stress, mainly in the central nervous system (CNS), which loses detoxification potential upon aging. In fact, aging is the main risk factor in the two most prevalent neurodegenerative diseases in Western societies with high life expectances: Parkinson’s (PD) and Alzheimer’s (AD) [1]. Unlike AD [2], PD patients have efficacious therapies that address symptoms but do not result in neuroprotection, i.e. they not impede disease progression [3,4]. 3, 5, 4′-trihydroxy-stilbene, commonly known as resveratrol, is among the most promising natural compounds with neuroprotective potential. Interest in resveratrol came from noticing that part of the benefits Mediterranean diet were due to consumption of wine, which is considered the main natural source of the compound. The natural molecule was first identified in 1939 by a Japanese researcher, Michio Takaoka, after extraction and purification from a medicinal herb, *Veratrum album var grandiflorum* [5,6]. Resveratrol naturally exists in *cis*- and *trans*-configurations although *trans*-resveratrol (RSV) seems to be the most biologically active isomer.

Two courses of action are designed to assess neuroprotection of RSV, one, epidemiologically based, consists on longitudinal studies of cohorts drinking wine in a daily basis. Nevertheless, RSV intake in “chronic” but moderate consumption of wine is probably not enough to boost innate antioxidant mechanisms. In this direction, a combination of different natural products in daily diets [up to 337 polyphenols are reported in a French population of circa 5000 participants with a total intake of approximately 1 g/day; cohort SUpplémentation en VItamines et Minéraux AntioXydants (SU.VI.MAX)], [7], may have an impact on reducing oxidative stress. The second approach is to assess the effect of RSV on nerve cells as a dietary supplement and at concentrations that are comparatively higher than those found in wine. Special care in such studies has to be taken in converting doses tested in rodents to doses in humans [8]. A placebo-controlled, double-blind trial has even showed that RSV administration to mild-to-moderate AD patients is safe and modifies some of the validated AD biomarkers [9].

The neuroprotective actions of RSV have been studied in both *in vivo* and *in vitro* models of AD [reviewed in [10]]. The results in animal models have been always promising despite the limited knowledge on how to afford neuroprotection and the limited knowledge of the molecular basis of RSV benefits. The antioxidant potential of RSV does not seem the cause of its beneficial effect as deduced from the trials on "antioxidant and Alzheimer’s" registered in *ClinicalTrials*.*gov* a US-National Institutes of Health-dependent office. With the exception of vitamin E, none of the tested compounds of this study (coenzyme Q, fish oil or an ultrafiltrate of animal blood that has to be intravenously administered) led to positive results. The potential benefit of vitamin E did not exceed the effect of the main drug prescribed to AD patients, memantine (an allosteric modulator of NMDA receptors). In fact, despite its “antioxidant” power, vitamin E may even be a pro-oxidant for plasma lipoproteins [11,12]. Results were unfortunately more deceiving on testing antioxidants to combat PD as 24 trials gave negative results. Parkinson’s^UK^ (https://www.parkinsons.org.uk) indicates: “*Excessive amounts of antioxidant vitamin supplements can adversely affect your health and wellbeing*, *and may interfere with your Parkinson's medication*”. Based on the hypothesis of antioxidants as neuroprotective agents, a further issue is the need for these compounds to reach the CNS with fairly intact antioxidant capabilities. It is unlikely that RSV penetrates the brain with significant antioxidant power. The aim of the present study was to analyze genomic data from experiments with rodents taking RSV in their diet and to identify genes that may mediate the reported beneficial effects of RSV in the CNS.

## Results

### Effect of RSV in gene expression

**Fig 1:**
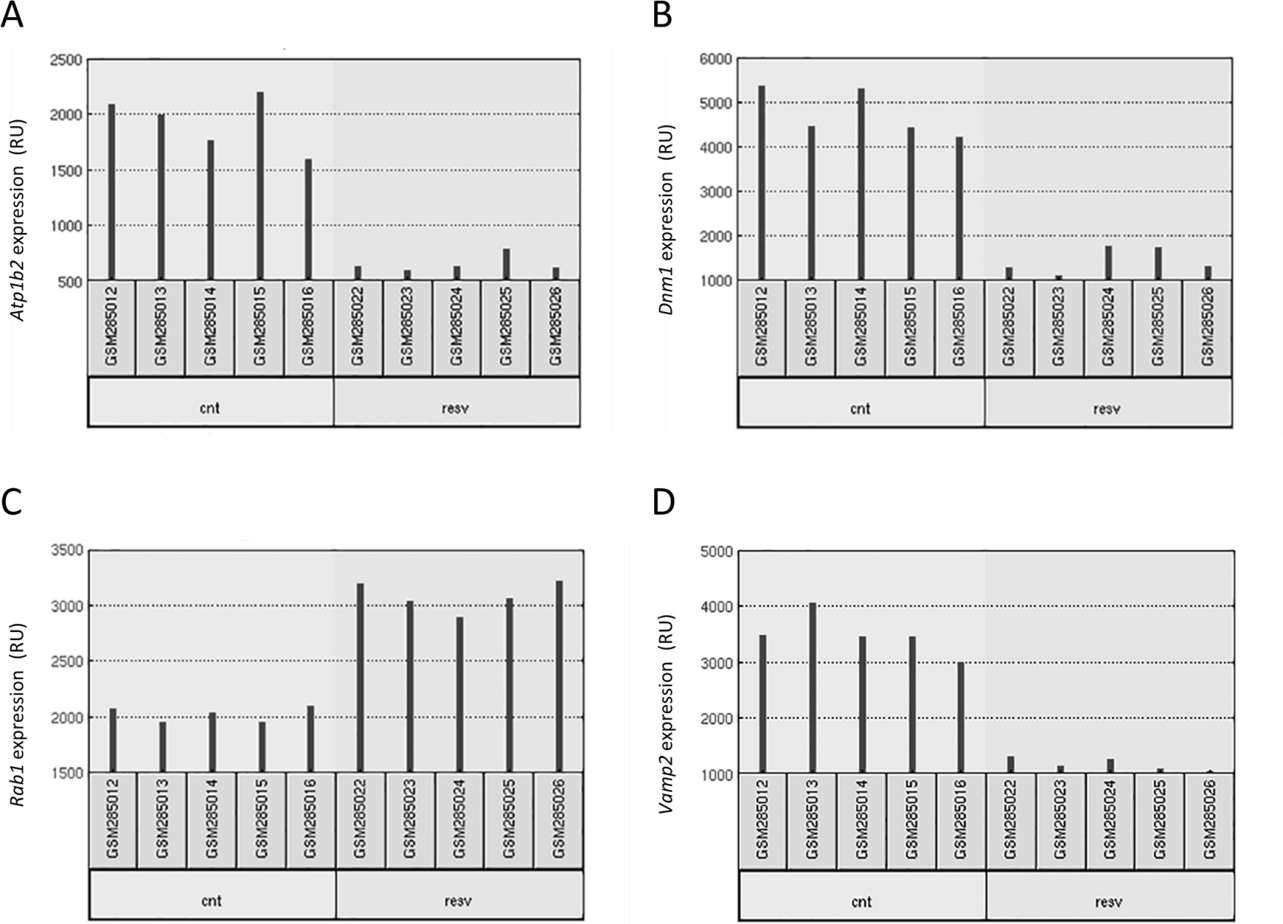
Raw data for four different genes: *Atp1b2* (A), *Dnm1* (B), *Rab1* (C) and *Vamp2* (D). Each red bar represents, in relative units (RU), the expression measurement from a sample. The five replicates correspond to five different animals (under control or under RSV-containing diets). (source: http://www.ncbi.nlm.nih.gov/geo/geo2r/?acc=GSE11291).

**Fig 2:**
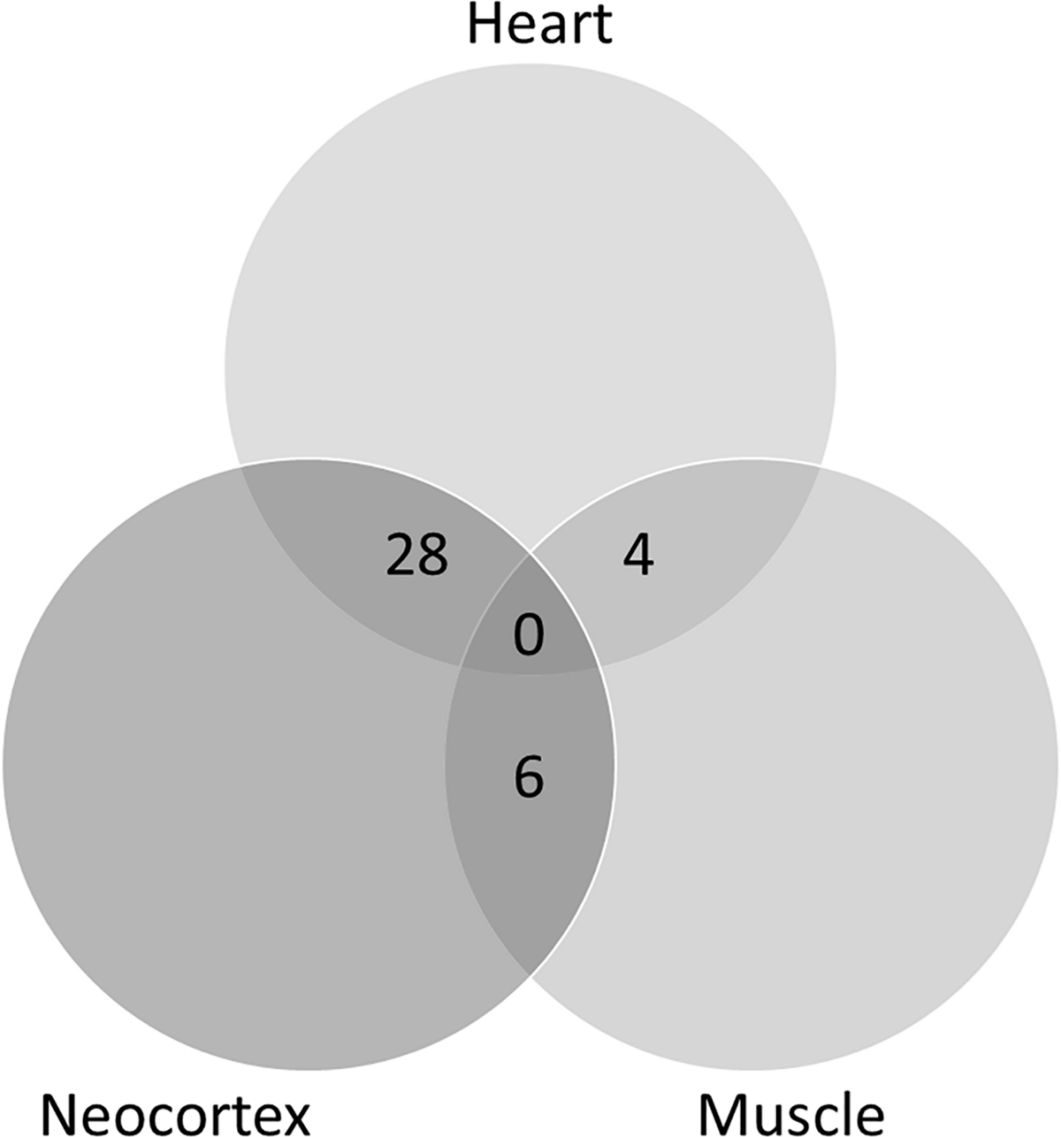
Venn diagram constructed with lists of differentially expressed genes corresponding to the three tissues (neocortex, heart and muscle) as identified using the GEO2R tool. The lists of genes were first curated as indicated in Material and Methods.

The main objective of this work was to study whether RSV affects gene expression in the brain and how this can be related with neuroprotection in neurodegenerative diseases. A previous analysis was performed taking advantage of similar data available from experiments in two other tissues, skeletal muscle and heart [13]. Therefore, our first aim was to analyze whether the effect of RSV in gene expression impacted on the same genes in the three different tissues (neocortex, skeletal muscle and heart). The analysis was conducted using GEO2R with default settings (see Methods), which provided a list of 250 differentially expressed genes for each tissue. An example of raw data for four of the genes differentially expressed upon RSV intake is shown in Fig 1. Lists were compared and a Venn diagram was constructed, as shown in Fig 2. Using a cut-off of change in gene expression of two-fold no gene could be found in common as differentially expressed in all three tissues. The significance level of pairwise comparison was 0.05 according to the Fisher test. Therefore, it appears that RSV affects the expression of different genes in different tissues.

### RSV and differential gene expression in the CNS

When comparing the expression in neocortex from mice taking or not RSV in their diet, we selected those genes that were up or down by two-fold and with a p<0.05 (adjusted, Benjamini & Hochberg -false discovery rate- test). Analysis of gene expression using GeneSpring provided a list of genes whose number varies according to the selected parameters (Table 1). The list of the 149 genes selected according to the criteria of two-fold cut-off and p<0.05 is provided as S1 Table. The list of the 250 genes selected by GEO2R according to default options, i.e. Benjamini & Hochberg false discovery rate adjustment to the p-values, auto-detect log transformation and NBCI-generated platform annotation to display results and to the criteria of higher statistical potency (lower p-values thus providing more confidence in the effect of RSV) is provided as S2 Table. The R script is provided in S2 Text. Fold-change values from the two lists are slightly different because list summarization by GeneSpring is performed combining intensity values from different probes of a given gene in the probe set, to get a simple intensity value for each gene. The default list obtained by GEO2R ranks gene expression according to the statistical significance (-increasing- P value) of the fold change calculated for each individual probe. Hence, fold-changes in GEO2R do not correspond to the expression of genes but to the expression of individual probes.

**Table 1:** Number of differentially expressed genes detected by GeneSpring depending on p-value and absolute fold change (FC) values. Analysis of data, from control- and RSV-enriched diet both in quintuplicates, were performed using moderated T-test, the multiple testing correction of Benjamini-Hochberg and asymptotic p-value (adjusted) computation.

	p all	p < 0.05	p < 0.02	p < 0.01	p < 0.005	p < 0.001
**FC all**	45101	8684	6166	4729	3623	1782
**FC > 1.1**	19195	8684	6166	4729	3623	1782
**FC > 1.5**	1338	1275	1232	1192	1133	903
**FC > 2.0**	158	149	148	145	144	129
**FC > 3.0**	6	6	6	6	6	6

**Fig 3:**
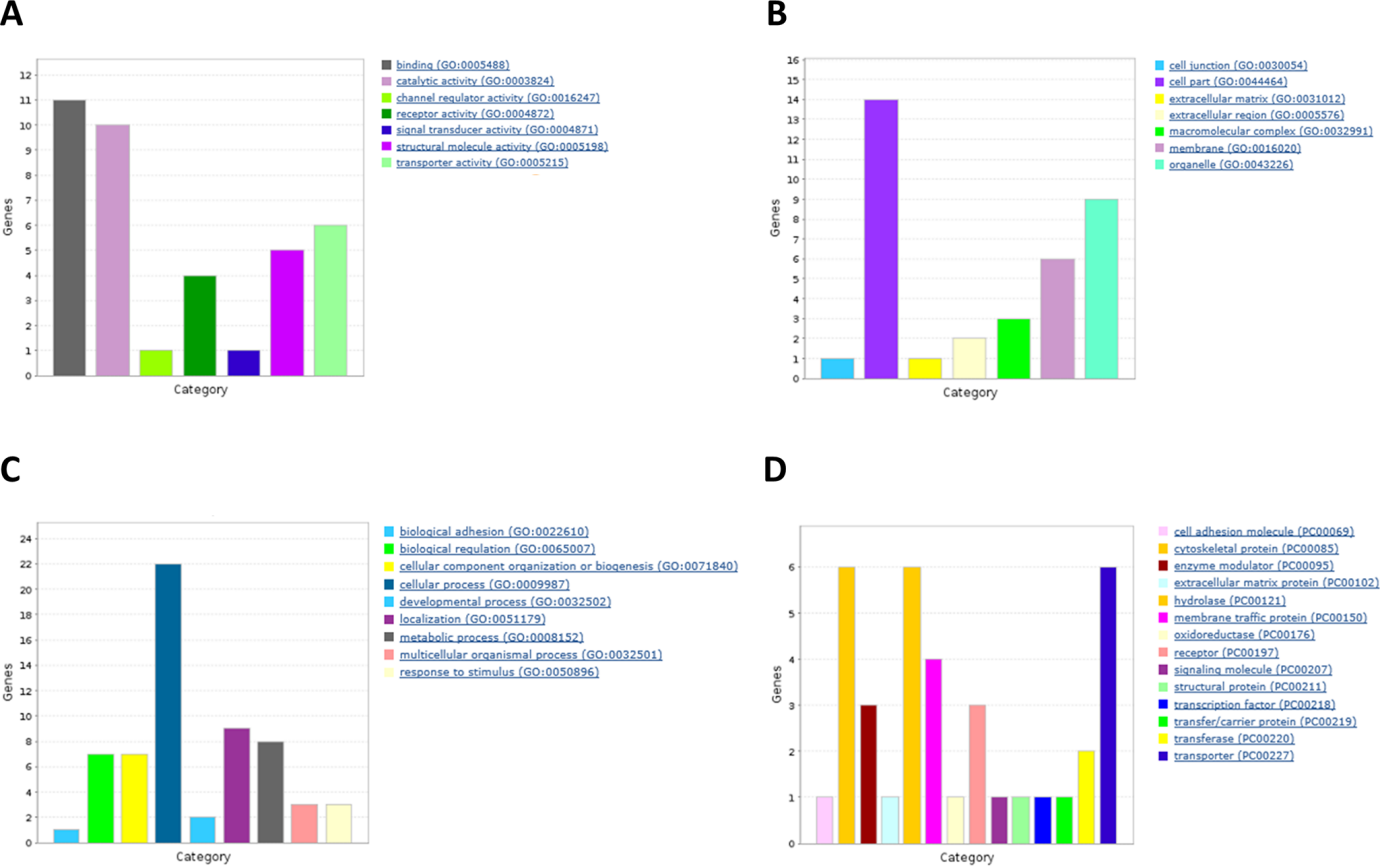
Gene Ontology bar charts for three different criteria: biological process (A), cell localization (B) and molecular function (C). The list of differentially expressed genes after RSV diet in neocortex were classified by Gene Ontology (GO) and drawn using Panther (www.pantherdb.org/)

We used a Venn diagram to look for genes present in both lists (from GEO2R and GeneSpring analysis). Curating the list of coincident genes (see Methods), we selected 26 for further study. As shown in Table 2, most genes (22 out of 26) were down-regulated in the neocortex of animals treated with RSV. With these 26 candidates we used panther (www.pantherdb.org/) to draw Gene Ontology bar charts based on three different criteria: biological process, cell localization and molecular function (Fig 3). It should be noted that the percentage of down-regulated genes was even higher (91%) in the non-curated list.

**Table 2:** Selected differentially expressed genes.

**Gene Symbol**	**Gene Title**	FC-G2R*	FC-GS*	**Expression**
*Strap*	Serine/threonine kinase receptor associated protein	1.653	3.105	Up
*Pkp4*	Plakophilin 4	1.579	2.166	Up
*Rab2a*	Member RAS oncogene family	1.518	2.413	Up
*Cpne3*	Copine III	1.345^a^	2.167^a^	Up^a^
*Actn1*	Actinin, alpha 1	0.422	0.496	Down
*Arf3*	ADP-ribosylation factor 3	0.418	0.456	Down
*Atp6v01*	ATPase, H^+^ transporting, lysosomal V0 subunit A1	0.512	0.489	Down
*Atp1a3*	ATPase, Na^+^/K^+^ transporting, alpha 3 polypeptide	0.213	0.210	Down
*Atp1b2*	ATPase, Na^+^/K^+^ transporting, beta 2 polypeptide	0.343	0.389	Down
*Cacng7*	Calcium channel, voltage-dependent, gamma subunit 7	0.365	0.409	Down
*Crtc1*	CREB regulated transcription coactivator 1	0.456	0.460	Down
*Dbn1*	Drebrin 1	0.296	0.355	Down
*Dnm1*	dynamin 1	0.305	0.344	Down
*Epn1*	Epsin 1	0.464	0.472	Down
*Gfap*	Glial fibrillary acidic protein	0.475	0.472	Down
*Hap1*	Huntingtin-associated protein 1	0.490	0.475	Down
*Mark4*	MAP/microtubule affinity-regulating kinase 4	0.062	0.460	Down
*Rab5b*	Member RAS oncogene family	1.518	0.484	Down
*Nrxn2*	Neurexin II	0.330	0.370	Down
*Ogt*	O-linked N-acetylglucosamine (GlcNAc) transferase (UDP-N-acetylglucosamine-polypeptide-N-acetylglucosaminyl transferase	0.377	0.370	Down
*Palm*	Paralemmin	0.438	0.494	Down
*Ptprn2*	Protein tyrosine phosphatase, receptor type, N polypeptide 2	0.375	0.393	Down
*Ptprs*	Protein tyrosine phosphatase, receptor type, S	0.359	0.450	Down
*Syn2*	Synapsin II	0.464	0.471	Down
*Timp2*	Tissue inhibitor of metalloproteinase 2	0.436	0.442	Down
*Vamp2*	Vesicle-associated membrane protein 2	0.336	0.343	Down

* Resveratrol-enriched versus control diet. FC-G2R: Fold Change according with GEO2R. FC-G2R: Fold Change according with GeneSpring.

^a^ Despite different FC values from GEO2R and GeneSpring analysis, the gene is upregulated.

Among molecular function, protein binding was the most represented group with 31% of the genes, followed by catalytic activity (26%) and structural function (15%). With respect to biological processes, cell communication (26%) was the most important biological process followed by metabolism (16%) and localization (18%). Finally, regarding cell localization, the majority of gene products were in intracellular organelles.

**Fig 4:**
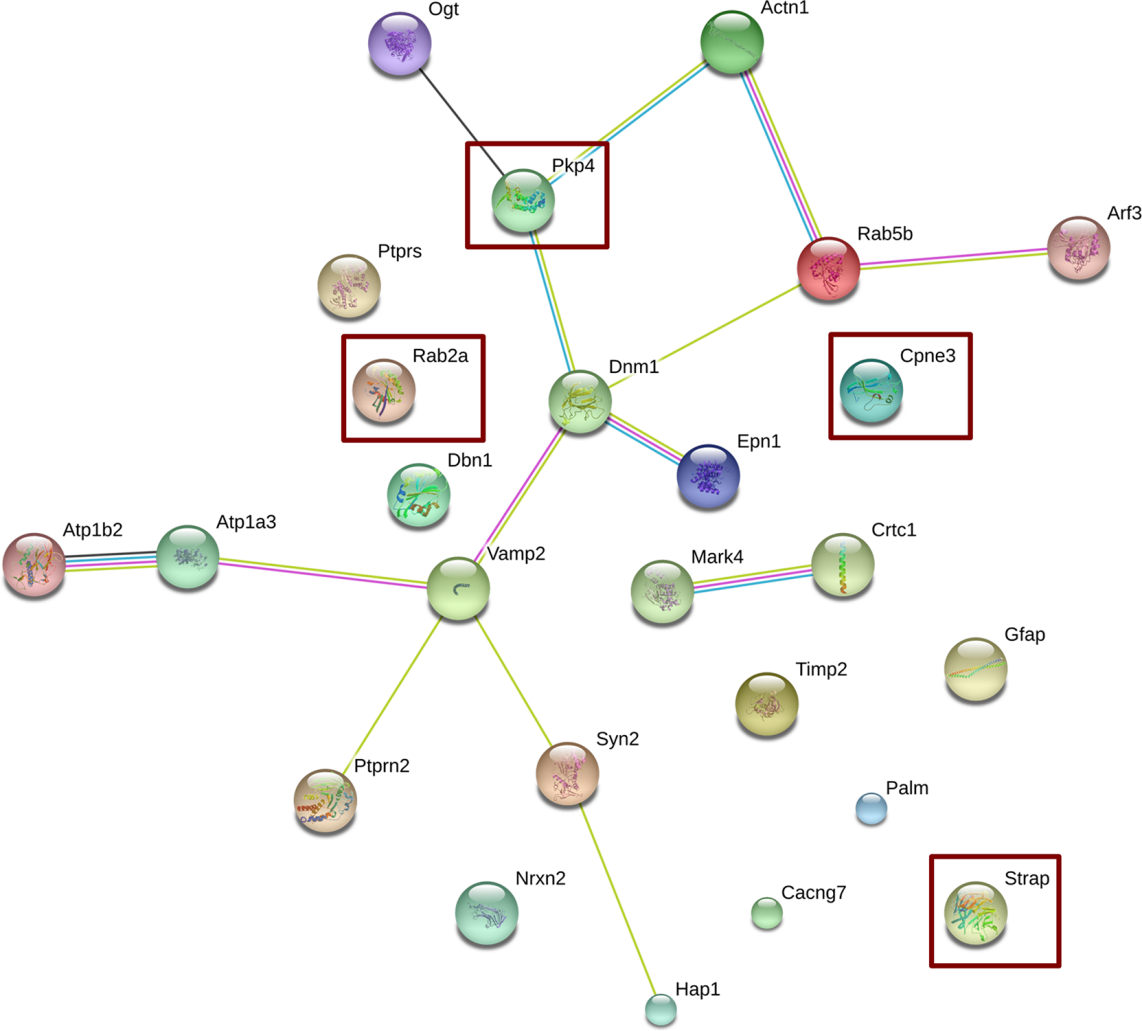
Network analysis using STRING. The list of differentially expressed genes in neocortex after RSV diet was subjected to Network analysis using the STRING software as described in Methods. Square interaction score was set at medium confidence (0.4; scores range from highest, 0.9, to low, 0.15, confidence). Other selected parameters were: average node degree: 1.12, local clustering coefficient: 0.32 and active interaction sources including: text mining, experiments, databases, co-expression, neighborhood, gene fusion and co-occurrence. Moreover, no restrictions were forced in the number of interactions to show. Overexpressed genes are shown within red rectangles. The colors of the edges represent the different types of protein associations, either from known or predicted interactions: from curated databases (blue), experimentally determined (magenta), text mining (green) and co-expression (black).

Noteworthy, further relevant information was obtained by using STRING (STRING CONSORTIUM 2016), a public available tool that allows the generation of functional protein association networks. The resulting network, taking into account all genes displayed in Table 2, is shown in Fig 4. Remarkably, the low protein-protein interaction enrichment p-value (2.27 e^-29^) indicated significantly more interactions than expected from a random set of proteins of similar size, drawn from the genome, meaning that the products of the identified genes are most likely biologically connected. The following genes did not display connections with other genes: *Ptprs*, *Rab2a*, *Dbn1*, *Cpne3*, *Timp2*, *Palm*, *Cacng7*, *Strap* and *Gfap*. Apart from a marginal but interesting connection between *Mark4* and *Crtc1*, all other genes were connected via two main nodes, namely *Dnm1* and *Vamp2*. These two genes were interrelated and, whereas *Dnm1* establishes direct links with *Pkp4*, *Rab5b* and *Epn1*, and indirect links with *Ogt*, *Actn1* and *Arf3*, *Vamp2* establishes direct links with *Atp1a3*, *Syn2*, *Ptprn2*, and indirect ones with *Atp1b2* and *Hap 1*. For comparison purposes a network constructed using the non-curated list is provided in S1 Fig. When the STRING analysis was performed using the non-curated list, we basically found the same main hubs, *Dnm1* and *Vamp2*, although the number of non-connected genes was much higher and this fact reinforces the appropriateness of list curation. However, in the case of *Dnm1* the connection through *Epn1* was further enriched with *Aes* and *Atp6v0a1*; and in the case of *Vamp2*, the interaction with *Syn2* was further connected with an additional hub, *Dlg4*. The PPI enrichment p-value for this list was higher than the one obtained with the curated list, 2.73 e^-09^, but still showing that this network has significantly more interactions than expected from random assignments using proteins of similar size drawn from the genome.

### Effects of acute RSV treatment in glioma C6 cells

**Fig 5:**
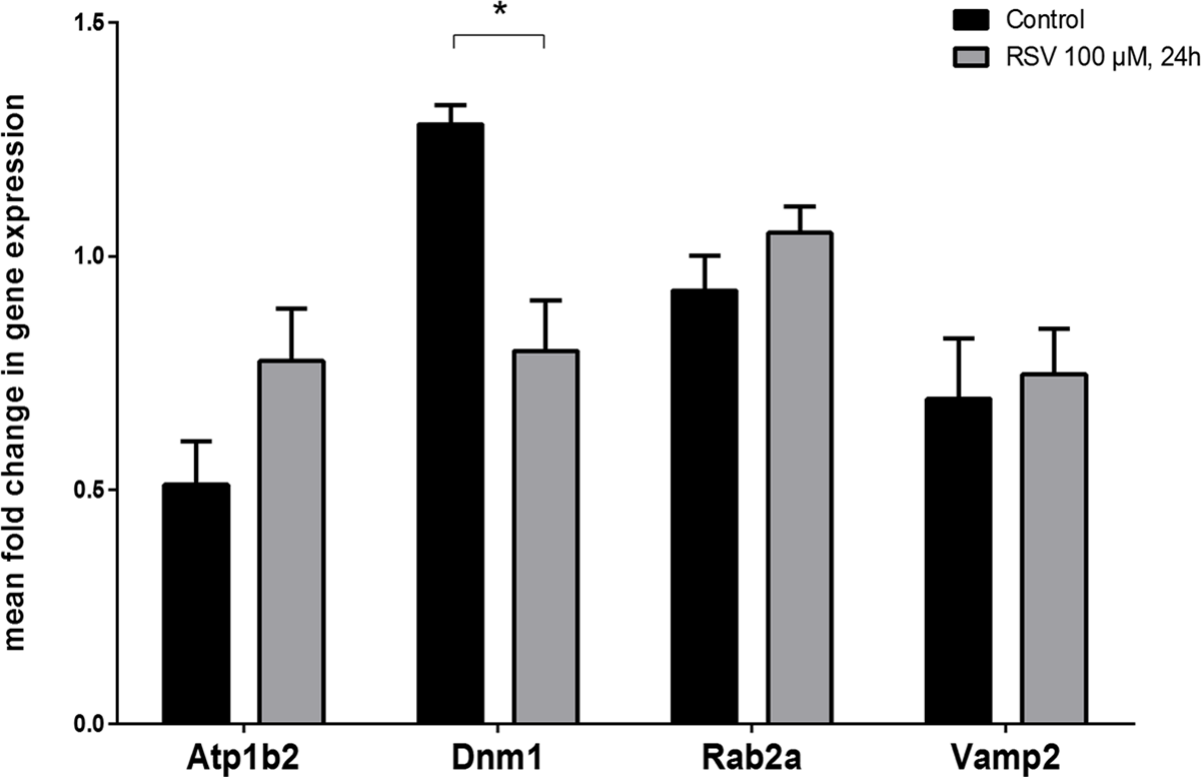
Gene expression evaluated by reverse transcription, quantitative real-time PCR (RT-qPCR) in control and in 100 μM RSV-treated C6 glioma cells. mRNA samples isolated from cells incubated for 24 h were analyzed by RT-qPCR using specific probes for *Atp1b2*, *Vamp2*, *Rab2a*, and *Dnm1*. β-actin mRNA was used as control. Data are mean ± SEM of four independent experiments. * p < 0.05 according to Student’s t test.

Neocortex is composed of a variety of neuronal and glial cell types. Therefore, the differential expression of the 26 candidate genes may occur in different CNS cells. Based on the data of RSV effect in neocortex and as neuronal cells of the adult mammal CNS are more refractory than glial cells to early changes in gene expression [14–16] we treated a glial cell line, C6, either in the absence (control group) or in the presence of 100 μM RSV for 24 h. Specific mRNA expression for 5 genes selected from the list in Table 2, *Atp1b2*, *Dnm1*, *Rab2a* and *Vamp2*, was determined by Real-Time qRT-PCR (see Methods). *Dnm1* and *Vamp2* were selected because they were nodes having more edges in Fig 5. *Atp1b2* was selected because genes related to energy-homeostasis are very relevant in glia-neuron interactions, and *Rab2a* was selected because it is similarly important for vesicular fusion and trafficking as *Vamp2*. The mRNA for *Syn2* was also analyzed as a control for a gene not expressed in glia (its mRNA was not detectable in glioma C6 cells). As seen in Fig 5, acute RSV treatment did not induce changes in the expression of *Rab2a* or *Vamp2*, whereas it induced a non-statistically significant increase in *Atp1b2* and, remarkably, led to a statistically significant down-regulation of *Dnm1*.

## Discussion

A first aim of this work was to compare the effect of RSV on gene expression in three different tissues. Tissues came from the same animals, C57BL/6xC3H/He mice, which received equally similar caging and environmental conditions. Moreover, the genomics methodology was identical and, therefore, the results are fully comparable. RSV affects gene expression in a different way in heart, neocortex and skeletal muscle, i.e. the effect was tissue-specific. Also of interest is highlighting that this differential effect in the three tissues does not cause major side effects and, in fact, RSV is a safe compound that may be taken at high doses without significant phenotypic alterations. Safety in animals agrees with the safety of the compound shown in a clinical trial involving mil-to-moderate AD patients [9].

The main objective of the present work was to take advantage of genomics data to understand how consumption of RSV is affecting CNS functionality. No gross alterations in gene expression were expected since RSV-consuming animals did not display any particular neural-related phenotype (peripheral or central) [13]. Indeed, the expression of several genes was affected but the fold change, i.e. the expression in RSV-taking animals *versus* the expression in animals taking the standard diet was relatively small. In this regard, 22 out of the 26 selected genes whose expression is significantly affected by the use of RSV are down-regulated. Therefore, it seems that the reported beneficial effect of RSV in CNS alterations [see [17] for review] correlates with simultaneous reduction of the expression of different genes. Network analysis showed that among interconnected genes (under moderate stringent parameters) all but *Pkp4* were down-regulated. The three main nodes in the network constituted by 14 genes (Fig 4) were *Pkp4 Dnm1* and *Vamp2*; *pkp4* and *Dnm1*, and *Dnm1* and *Vamp2*, which were connected in a direct way (Fig 4). Overall it seems that up-regulation of *Pkp4* drives the down-regulation of *Dnm1* and *Vamp2*, which in turn drives the down-regulation of the other 11 genes in the network. This hypothesis is further supported by the fact that *Pkp4* is the only upregulated gene in the network, and by the finding that all genes directly connected to *Pkp4 (Dnm1*, *Actn1* and *Ogt*) are down-regulated. The product of *Pkp4*, plakophilin 4 or p0071, an armadillo/catenin family member, is relevant for cytoskeletal organization [18] and seems to be expressed in both neurons [19] and glia [20]. *Dnm* gene products, dynamins, are large GTPases that polymerize and contribute to scission and fission of vesicles from membranes being their principal function related to endocytosis and vesicle transport. In particular, *Dnm1* is heavily expressed in brain and also acts as microtubule-, and phospholipid-binding protein [21,22]. The product of *Vamp2*, synaptobrevin-2, a member of the vesicle-associated membrane protein (VAMP)/synaptobrevin family, is widely expressed in the CNS [23], interacts with synaptophysin and regulates *inter alia* pre-synaptic vesicle traffic [24] and neuronal morphogenesis and the branching of axons [25]. In summary, the protein product of up-regulated *Pkp4* and most of the down-regulated genes in the network take part, directly or indirectly, in events related to the cytoskeleton and to the transport of subcellular components. Some of the functions of gene products in Table 2 focus on the synapse, specifically in the mobilization of the presynaptic vesicles and the fusion of those with the presynaptic membrane and the subsequent neurotransmitter release.

Regarding the potential benefits of RSV consumption in AD therapy, literature indicates that this phytochemical affects in a neuroprotective mode three pathophysiological mechanisms of the disease: it reduces i) the pathology caused by β-amyloid peptide [26–30], ii) oxidative stress [31] and iii) neuroinflammation [32–34]. It should be also noted that RSV modulates the expression of genes involved in cytoskeletal structure and vesicular traffic in soma, neuronal axon/ramifications and nerve terminals, including synaptic pruning, something that does not occur in heart or muscle [35]. Importantly, our results do not favor antioxidant actions due to RSV. It is unlikely that an antioxidant-behaving molecule mainly affects cytoskeleton structure and/or vesicular traffic and synaptic events.

Three of the four genes that were upregulated, *Rab2a*, *Strap* and *Cpne3*, and 6 down-regulated genes, *Gfap*, *Ptprs*, *Dbn1*, *Timp2*, *Palm*, *Cacng7*, do not appear interrelated in the constructed network. The lack of connections among these genes may reflect underscoring glial genes due to the higher qualitative and quantitative research focused on neuronal genes. In fact, the lack of interconnections in the case of *Gfap*, whose product is the glial fibrillary acidic protein, i.e. a glial protein, together with the finding that *Dnm1* is differentially expressed in C6 cells treated with the phytochemical (Fig 5), suggest that glia could have a significant role in mediating the effect of RSV. Furthermore, exosomes, which appear as important in brain glial-neuronal networks [36] may be produced by glia in a process in which some of the differentially expressed genes found in this study are involved. An example is copine 3, a product of *Cpn3*, that in our opinion deserves attention in the CNS for being ubiquitous and for its role as a calcium-dependent phospholipid-binding protein [37,38]. To our knowledge there are no studies on *Cpn3* expression and function in neurons or glia.

Our last aim was to identify any significant gene whose expression would vary after an acute treatment with RSV. We hypothesized that acute changes in neuronal cultures would not be as informative and significant as acute changes in glial cells. Neuronal plasticity resulting in neuroprotective or memory-enhancing properties requires time and involves a myriad of glial-neuronal interactions [39]. In addition, any region in the CNS has more diversity in neuronal than in glial cell types. Thus, the study of RSV-induced changes in a given neuronal cell type may not be correlated to other neuronal cell types present in the same CNS area. The less specialized function of glial cells could serve to find an early event that may help to understand how glia participates in the effects of RSV on CNS. Hence, we used a well-established C6 glioma cell line [40], which has characteristics of stem cells such as multi-lineage differentiation and self-renewal [41]. Upon treatment with RSV, the expression of Dynamin 1 (*Dnm1)* was changed but not for other genes (Fig 5) whose expression was determined in parallel. The direction of the change was similar to that obtained upon chronic treatment in mice, i.e. the treatment with RSV decreased *Dnm1* mRNA levels. Since its discovery, cloning and release of expression data, the enrichment of *Dnm1* in CNS was attributed to neuronal expression. In our control using a neuronal-specific gene we demonstrated that glioma cells express the *Dnm1* gene and that its expression is affected by RSV, even in acute treatments. Of note is that another functional genomics study indicates that D*nm1* is expressed in glia-derived cell lines (NCBI-GEO, ref. GDS4296). In this sense, our RT-qPCR analyses indicate that glial *Dnm1* should be also taken into account to understand the overall mode of action of RSV in the CNS. Recent studies in *Drosophila melanogaster* have taken advantage of temperature sensitive alleles of *shibire*, whose product is a dynamin [42,43], to show that this glial protein is required for programmed axon pruning and stabilization of motor neuron branches during metamorphosis [44,45]. Even if there are very few studies of dynamins in mammalian neural cells, the work of Sakai *et al*. (2013) is remarkable as it shows that *Dnm1* is expressed in microglia and regulates the activity of voltage-gated ion channels [46]. Taken all these data together, it appears that the role of dynamin 1 as a central node in the network of differentially expressed genes (Fig 4) may be due to neuronal dynamin 1 even though the glial gene must also be taken into account.

Finally, our findings detect a decrease in the expression of CREB-regulated transcription coactivator 1 (Crtc1) upon RSV consumption. This result suggests that the benefits on this compound in diseases with cognition deficits might be by CREB-independent mechanisms. On the one hand, it is well established that activation of CREB by phosphorylation leads to synaptic plasticity and has memory-enhancing properties [47]. On the other hand, RSV may revert the cognition impairment in chronic unpredictable stress conditions [48]. Taken together, these results indicate that either memory-enhancer properties of CREB could be independent of *Crtc1* levels or chronic consumption of RSV exerts memory-enhancing effects by a CREB/*Crtc1*-independent pathway. This latter hypothesis may fit with the beneficial effects of RSV intake in PD models; in which such benefits are not associated to CREB activation [49–51]. As above mentioned our results do not favor, as reported elsewhere Lu *et al*. (2008), neuroprotection against dopamine denervation in PD models due to antioxidant or radical scavenging properties of the compound [52].

Despite limitations in this type of study, namely heterogeneity of cell type in neocortex, limited number of verified genes, usage of a glioma cell line that is different from real glial cells present in the brain, it is tempting to speculate that RSV achieves a modulation of gene expression in the CNS that positively affects the life span and the functionality of nerve cells by default mechanisms common to many of them. The products of the genes in Table 2 may serve as a good starting point for the study of neuroprotective effects of RSV and the role of neuron-glia interactions in different neurodegenerative diseases and to look for transversal mechanisms of neuroprotection.

## Material and methods

### Data retrieval

Data were searched within the Gene Expression Omnibus (GEO) database under “GEO DataSets” (https://www.ncbi.nlm.nih.gov/gds/) using as keywords: resveratrol and neocortex, selecting the only hit, i.e. the report by Barger et al., [13] with reference ID “GSE11291” (www.ncbi.nlm.nih.gov/geo/query/acc.cgi?acc=GSE11291) which refers to a study where the design consisted in feeding male (C57BL/6xC3H/He) F1 hybrid mice from 14 to 30 months of age with either a control diet, a RSV-containing diet or a calorie restricted diet [13]. In the present study, these data were analyzed using two different software tools: GEO2R and GeneSpring (see “Data analysis tools” section). Retrieved data resulted from gene expression determined using Affymetrix Mouse Genome 430 2.0 arrays, the specific platform used was GPL 1261, containing probes for 45,000 genes. The profiling was assayed in samples from heart, skeletal muscle and brain (neocortex) [13]. The control diet provided 84 kcal whereas the calorie restricted one provided 63 kcal per mouse and week. RSV supplement (4.9 mg/Kg and day) was given to animals taking the control diet. At 30 months of age, mice were sacrificed and tissues were collected, flash-frozen in liquid nitrogen and stored at -80°. Functional genomics experiments were performed with these samples. For each condition, five replicates were processed and the results for 4 genes are shown in Fig 1. Representative graphs show that the five replicates have small standard deviation in both conditions, either from animals taking control diet or from animals taking a RSV-containing diet.

### Data analysis tools

GEO2R (www.ncbi.nlm.nih.gov/geo/info/geo2r.html) was used to perform comparisons on GSE11291 data using GEO query and limma (Linear Models for Microarray Analysis) R packages from the *Bioconductor* project (www.bioconductor.org). The approach for false positive detection is reported elsewhere [53]. It should be noted that GEO2R has a feature that checks the character of values and, if required, it automatically performs a base 2 log transformation. Raw results are shown in S1 Table, which lists 250 differentially expressed genes ranked by p-value.

For comparison purposes we also used GeneSpring, which is a commercially-available software package specially designed for analysis of microarray data [54]. By retrieving the genomics data from control- and RSV-enriched diet both in quintuplicates (10 data files in total), and by subsequent analysis using RMA (robust microarrays average) summarization algorithm, baseline to median of control samples, a fold change cut-off higher than 2, moderated T-test, the multiple testing correction of Benjamini-Hochberg and asymptotic <0.05 p-value (adjusted) computation, we obtained 149 differentially expressed genes (S2 Table and S1 Text).

The raw lists of differentially expressed genes were manually curated. Genes not selected for further studies were those whose physiological role is not known and/or whose cell/tissue function is not established. First of all, genes whose products are suspected due to homology to genes from other species (e.g. *Drosophila*) were discarded. Examples are: *Ap2a1* (the gene product is: adaptor-related protein complex 2, alpha 1 subunit), *Actl6b* (the gene product, actin-like 6B, shares sequence homology with actin with no defined function) and *Bicd2* (the gene product is: bicaudal D homolog 2 of *Drosophila*) or *Cnih2* (the gene product is: cornichon homolog 2 of *Drosophila*).

### Online tools for network construction

With a list of two-fold differentially expressed genes in samples from mice fed with/without RSV, it is possible to look for functional relationships among the gene products. Connections between gene products can be conceptualized as networks and the size and complexity of these networks present a unique opportunity to look the transcriptome as something more than just a static collection of data [55,56]. The “network view” of genes whose expression correlate in two given situations is increasingly being used in many areas of applied biology such as to increase the statistical power in human genetics, to aid in drug discovery, to close gaps in metabolic enzyme knowledge and to predict phenotypes and gene functions [55,56].

We took advantage of online resources to integrate both known and predicted interactions (Functional protein association networks). Interactions in STRING (Search Tool for the Retrieval of Interacting Genes) are provided with confidence scores and this tool also offers accessory information such as protein domains and 3D structures (with link to databases) if available. The version of the program accessed via STRING DB website (http://string-db.org. [55]) included > 2000 organisms, ranging from Bacteria and Archaea to humans. Selected parameters were: average node degree: 1.12 and average local clustering coefficient: 0.32. Medium confidence (0.4) was selected as square interaction score (scores range from highest, 0.9, to low, 0.15, confidence) and active interaction sources included were: text mining, experiments, databases, co-expression, neighborhood, gene fusion and co-occurrence. No restrictions in the number of interactions to show.

### Cell culture and RSV treatment

Rat C6 glioma cells were used to study gene expression as they have been previously used as cell model for the treatment of RSV and have showed to be able to uptake this polyphenol [56]. Rat C6 glioma cells were obtained from the American Type Culture Collection (ATCC, USA). The cell clone was originally developed from a N-nitrosomethylurea-induced rat glial tumor [57]. Cells were grown, as described elsewhere [58], in DMEM (Dulbecco´s modified Eagle´s medium) supplemented with 10% fetal calf serum, 2 mM L-glutamine, 1% non-essential amino acids and antibiotics, in a humidified atmosphere of 95% air and 5% CO_2_ at 37°C. Cells in complete medium were incubated in the absence or in the presence of 100 μM RSV (Sigma Aldrich, Madrid, Spain) for 24 h as previously used by other authors using the same cell line [59]. At this concentration, RSV have previously showed neuroprotection against oxidative stress [60]. Dilutions from a concentrated RSV solution in ethanol were used. The final concentration of ethanol in the samples (both control and RSV-treated) was 0.4% v:v)

### Total RNA isolation and preparation of cDNA

Total RNA was extracted from C6 glioma cells using ABI 6100 Nucleic Acid PrepStation and chemicals according to the manufacturer’s protocol (Applied Biosystems, Foster City, CA). RNA purity was assessed by the 260:280 nm absorbance ratio; it was in the range 1.9–2.1. RNA concentrations were determined from the absorbance at 260 nm. Total RNA was stored at -80°C. One microgram of total RNA was reverse transcribed using Applied Biosystems’ High-Capacity cDNA Archive Kit according to the manufacturer’s protocol (Applied Biosystems, Foster City, CA).

### Quantitative real time PCR analysis

To assess relative gene expression in both control and RSV treated rat C6 glioma cells, quantitative real time-PCR (RT-qPCR) analysis was performed with an Applied Biosystems Prism 7500 Fast Sequence Detection System, using TaqMan universal PCR master mix according to the manufacturer’s specifications (Applied Biosystems, Foster City, CA). The TaqMan probes and primers for Atp1b2 (assay ID: Rn00569739-m1), Vamp2 (assay ID: Rn01465442_m1), Rab2a (assay ID: Rn00581858_m1), Dnm1 (assay ID: Rn00589865_m1) and β-actin (assay ID: Rn00578826_m1) were assay-on-demand gene expression products (Applied Biosystems, Foster City, CA) as previously described [61]. β-actin was used as endogenous control for normalization.

### Statistics and data analysis of PCR assays

Data are mean ± SEM of four independent experiments. Statistical data analysis was performed using Student t-test with the GraphPad Prism 6 program (GraphPad Software, San Diego, CA, USA). Differences between mean values were considered statistically significant at p<0.05.

## Supporting information

S1 Table Differential gene expression (resveratrol versus control diet) in neocortex by GeneSpring analysis. (DOCX)

S2 Table Differential gene expression (resveratrol *versus* control diet) in neocortex by GEO2R analysis. (DOCX)

S1 Text Data analysis for S2 Table. (DOCX)

S2 Text R script provided by GEO2R analysis. (DOCX)

S1 Fig Network analysis using STRING and the non-curated list of differentially expressed genes in neocortex after RSV diet. (DOCX)

## Abbreviations

AD: Alzheimer’s disease
CNS: Central Nervous System
Crtc1: CREB-regulated transcription coactivator 1
GO: Gene Ontology
PD: Parkinson’s disease
RSV: *trans*-resveratrol
RT-qPCR: quantitative real time polymerase chain reaction after reverse transcription
STRING: Search Tool for the Retrieval of Interacting Genes
