# Supplementary material for: A genomics approach identifies selective effects of trans-resveratrol in cerebral cortex neuron and glia gene expression
Source: PLoS One. 2017 Apr 25;12(4):e0176067. doi: 10.1371/journal.pone.0176067 (PMC5404873; doi:10.1371/journal.pone.0176067)
Supplement: S1 Table — (DOCX) [file pone.0176067.s001.docx]

**S1 Table. Differential gene expression (resveratrol *versus* control diet) in neocortex by GeneSpring analysis.**

| Probe ID | **p-value** | **Gene Symbol** | **Fold-Change*** | **Expression** |
| --- | --- | --- | --- | --- |
| 1419913_at | 0,00506982 | *Strap* | 3,105446 | Up |
| 1419918_at | 0,02155294 | *Tmed7* | 2,109502 | Up |
| 1419946_s_at | 0,01810246 | *Rab2a* | 2,412648 | Up |
| 1419975_at | 0,0206726 | *Scp2* | 2,8488476 | Up |
| 1420175_at | 0,04534128 | *Tax1bp1* | 2,6950142 | Up |
| 1426114_at | 0,00306318 | *Hnrnpab* | 2,0431983 | Up |
| 1436989_s_at | 0,00429173 | *Slc12a6* | 2,6196632 | Up |
| 1438677_at | 0,00869645 | *Pkp4* | 2,1664228 | Up |
| 1439005_x_at | 0,01184645 | *Ywhaz* | 2,0393016 | Up |
| 1449347_a_at | 0,01530217 | *Xlr4a///Xlr4b///Xlr4c* | 2,0207965 | Up |
| 1452259_at | 0,01103632 | *Phf20* | 2,3021722 | Up |
| 1419924_at | 0,03045716 | *Fnip1* | 2,1004384 | Up |
| 1430077_at | 2,56E-04 | *Srsf11* | 2,2512624 | Up |
| 1430675_at | 0,00584294 | *2900055J20Rik* | 2,3537824 | Up |
| 1430991_at | 0,04943238 | *1810014B01Rik* | 2,1294658 | Up |
| 1431094_at | 0,0029433 | *1110006E14Rik* | 2,3633192 | Up |
| 1435708_at | 0,0023245 | *Gls* | 3,0086899 | Up |
| 1436387_at | 0,0111481 | *C330006P03Rik///Homer1* | 2,9479864 | Up |
| 1436698_x_at | 6,62E-04 | *Tmem204* | 2,069954 | Up |
| 1437660_at | 0,00409557 | *Nktr* | 3,5034306 | Up |
| 1437883_s_at | 0,0030309 | *Pan3* | 2,0162847 | Up |
| 1438104_at^a^ | 9,04E-04 | *-----* | 2,3391836 | Up |
| 1438580_at | 0,03055207 | *Zcchc7* | 2,3795385 | Up |
| 1439090_at | 5,41E-04 | *Tbc1d23* | 2,4309156 | Up |
| 1439265_at^a^ | 0,00297553 | *-----* | 2,6256244 | Up |
| 1439972_at | 0,02623331 | *Etnk1* | 2,3959424 | Up |
| 1440020_at^a^ | 0,00535823 | *-----* | 2,0290642 | Up |
| 1440158_x_at^a^ | 0,03728085 | *-----* | 2,139802 | Up |
| 1441233_at^a^ | 0,00385156 | *-----* | 2,052111 | Up |
| 1441498_at^a^ | 0,03957317 | *-----* | 2,0071166 | Up |
| 1442491_at^a^ | 0,042584 | *-----* | 2,2585518 | Up |
| 1442849_at | 0,00181521 | *Lrp1* | 2,2587216 | Up |
| 1443166_at^a^ | 0,01110545 | *-----* | 2,4695823 | Up |
| 1444317_at | 0,01976994 | *Pcdh15* | 2,2296832 | Up |
| 1444488_at^a^ | 0,04051262 | *-----* | 2,0163538 | Up |
| 1452814_at | 0,01128769 | *Cpne3* | 2,1667027 | Up |
| 1458676_at | 0,01841348 | *Nktr* | 2,3161857 | Up |
| 1459746_at^a^ | 0,00723562 | *-----* | 2,0162017 | Up |
| 1415899_at | 0,03263804 | *Junb* | 2,0416157 | Down |
| 1416965_at | 5,75E-04 | *Pcsk1n* | 2,0779572 | Down |
| 1416997_a_at | 0,04149579 | *Hap1* | 2,1039934 | Down |
| 1417524_at | 8,99E-05 | *Cnih2* | 2,134673 | Down |
| 1417963_at | 0,00183608 | *Pltp* | 2,474894 | Down |
| 1418015_at | 0,00245628 | *Pum2* | 2,0038254 | Down |
| 1419580_at | 3,38E-04 | *Dlg4* | 2,2032099 | Down |
| 1419581_at | 4,70E-04 | *Dlg4* | 2,6981335 | Down |
| 1420575_at | 0,00141676 | *Mt3* | 3,1735036 | Down |
| 1420619_a_at | 2,00E-04 | *Aes* | 2,030584 | Down |
| 1420749_a_at | 3,06E-04 | *Pou6f1* | 2,180346 | Down |
| 1420752_at | 0,00370692 | *Dtx3* | 2,241042 | Down |
| 1420833_at | 3,60E-04 | *Vamp2* | 2,9129374 | Down |
| 1420924_at | 0,00121218 | *Timp2* | 2,2637916 | Down |
| 1420964_at | 6,27E-04 | *Enc1* | 3,0792174 | Down |
| 1421060_at | 0,0026607 | *Mllt1* | 2,4233067 | Down |
| 1421160_a_at | 6,65E-04 | *Rfng* | 2,0489209 | Down |
| 1421181_at | 0,00254407 | *Npcd///Nptxr* | 2,1517885 | Down |
| 1421267_a_at | 0,00137366 | *Cited2* | 2,278681 | Down |
| 1421368_at | 0,00112999 | *Scrt1* | 2,1797256 | Down |
| 1421789_s_at | 0,00297651 | *Arf3* | 2,1997707 | Down |
| 1422009_at | 0,00107605 | *Atp1b2* | 2,5733361 | Down |
| 1422034_a_at | 3,05E-04 | *Palm* | 2,0253801 | Down |
| 1422040_at | 0,00402003 | *Sema7a* | 2,083114 | Down |
| 1422119_at | 0,00171096 | *Rab5b* | 2,0656216 | Down |
| 1422321_a_at | 2,56E-04 | *Sf1* | 2,0105321 | Down |
| 1422564_at | 4,30E-04 | *Actl6b* | 2,154091 | Down |
| 1422733_at | 0,00861043 | *Fjx1* | 2,0518303 | Down |
| 1422799_at | 3,30E-04 | *Prrc2a* | 2,041788 | Down |
| 1423221_at | 0,00151931 | *Tubb4a* | 2,0138674 | Down |
| 1423561_at | 0,00342266 | *Nell2* | 2,3596766 | Down |
| 1425337_at | 0,00230888 | *Slc12a5* | 2,219729 | Down |
| 1425369_a_at | 0,00280509 | *Sox10* | 2,2581913 | Down |
| 1425724_at | 2,56E-04 | *Ptprn2* | 2,543627 | Down |
| 1425870_a_at | 0,00488991 | *Kcnip2* | 2,0496104 | Down |
| 1425963_at | 0,01065949 | *Cabp7* | 2,578839 | Down |
| 1426336_at | 0,00313826 | *Cacng7* | 2,446627 | Down |
| 1426508_at | 0,01463286 | *Gfap* | 2,118779 | Down |
| 1426617_a_at | 2,56E-04 | *Ttyh1* | 2,0057428 | Down |
| 1427039_at | 0,00112319 | *Epn1* | 2,1183033 | Down |
| 1427099_at | 5,75E-04 | *Maz* | 2,1732008 | Down |
| 1427385_s_at | 0,00760522 | *Actn1* | 2,0146441 | Down |
| 1427457_a_at | 0,007732 | *Bmp1* | 2,2051504 | Down |
| 1427481_a_at | 6,28E-04 | *Atp1a3* | 4,771516 | Down |
| 1427688_a_at | 0,00181521 | *Ptprs* | 2,222586 | Down |
| 1427754_a_at | 4,26E-04 | *Dnm1* | 2,9084404 | Down |
| 1427797_s_at | 9,22E-04 | *Ctse* | 2,0049634 | Down |
| 1428054_at | 9,63E-05 | *Slc8a2* | 2,3514955 | Down |
| 1428707_at | 2,00E-04 | *Ptms* | 2,208839 | Down |
| 1428708_x_at | 2,00E-04 | *Ptms* | 2,1431618 | Down |
| 1428740_a_at | 4,84E-04 | *Pigt* | 2,0762682 | Down |
| 1430437_a_at | 2,46E-04 | *Vps9d1* | 2,0253525 | Down |
| 1435026_at | 0,00125184 | *Spock2* | 2,1652362 | Down |
| 1435105_at | 0,00296834 | *Rnf208* | 2,0816808 | Down |
| 1435152_at | 0,00255062 | *Leng8* | 2,4380958 | Down |
| 1436665_a_at | 0,0023525 | *Ltbp4* | 2,0217338 | Down |
| 1436780_at | 0,00464696 | *Ogt* | 2,7064126 | Down |
| 1437491_at | 9,97E-04 | *Bicd2* | 2,0838335 | Down |
| 1439051_a_at | 3,18E-04 | *Mark4* | 2,1747503 | Down |
| 1449030_at | 5,52E-04 | *Syn2* | 2,1237392 | Down |
| 1450228_a_at | 0,00129144 | *Pip5k1c* | 2,1822708 | Down |
| 1451465_at | 0,00122605 | *Ubl7* | 2,209674 | Down |
| 1451734_a_at | 8,89E-04 | *Dbn1* | 2,81604 | Down |
| 1451834_at | 5,45E-04 | *Cacnb1* | 2,5373158 | Down |
| 1460385_a_at | 0,01176074 | *Rnf112* | 2,0208352 | Down |
| 1460650_at | 0,00160941 | *Atp6v0a1* | 2,044092 | Down |
| 1460724_at | 2,00E-04 | *Ap2a1* | 2,2571845 | Down |
| 1429009_at | 0,0162452 | *Snrnp70* | 2,0746307 | Down |
| 1430543_at | 4,44E-04 | *Clip3* | 2,0454984 | Down |
| 1433468_at | 0,00106769 | *Irf2bpl* | 2,0830867 | Down |
| 1434421_at | 0,00297651 | *Islr2* | 2,0600958 | Down |
| 1434908_at | 0,00169212 | *Scaf1* | 2,41717 | Down |
| 1435790_at | 0,00514465 | *Olfm2* | 2,037756 | Down |
| 1436485_s_at | 0,00231245 | *Whrn* | 2,030971 | Down |
| 1440390_at | 0,00663101 | *Fam171a2* | 2,1957946 | Down |
| 1441312_at | 8,89E-04 | *Cnnm1* | 2,2502968 | Down |
| 1442066_at | 3,06E-04 | *Pianp* | 2,5129974 | Down |
| 1443805_at | 0,00107605 | *Dact3* | 2,2227724 | Down |
| 1445502_at | 0,01411345 | *C78859* | 2,087152 | Down |
| 1446215_at | 0,00497132 | *Zfhx2as* | 2,3293736 | Down |
| 1455499_at | 0,0014724 | *Nrxn2* | 2,7064614 | Down |
| 1455701_at | 0,00112509 | *Arhgap33* | 2,6287336 | Down |
| 1456304_at | 0,00110311 | *Gm996* | 2,1405215 | Down |
| 1456476_at | 6,49E-04 | *Atxn2l* | 2,1301007 | Down |
| 1458443_at | 0,00191756 | *Crtc1* | 2,176137 | Down |
| 1459639_at | 0,00226227 | *Brsk2* | 2,160179 | Down |

* Resveratrol-enriched *versus* control diet

**^a^** No symbol assigned to this probe
