# Supplementary material for: A genomics approach identifies selective effects of trans-resveratrol in cerebral cortex neuron and glia gene expression
Source: PLoS One. 2017 Apr 25;12(4):e0176067. doi: 10.1371/journal.pone.0176067 (PMC5404873; doi:10.1371/journal.pone.0176067)
Supplement: S2 Table — (DOCX) [file pone.0176067.s002.docx]

| Probe ID  **S2 Table. Differential gene expression (resveratrol *versus* control diet) in neocortex by GEO2R analysis.** | **p-value** | **Gene Symbol** | **Gene Title** | | **Fold-Change*** | | **Expression** | |
| --- | --- | --- | --- | --- | --- | --- | --- | --- |
| 1456845_at | 0,00000188 | *Oxa1l* | | Oxidase assembly 1-like | | 4,26338694 | | Up |
| 1435708_at | 0,0000157 | *Th1* | | Th1 cytokine | | 3,44618464 | | Up |
| 1430077_at | 0,00000494 | *Srsf11* | | Serine/arginine-rich splicing factor 11 | | 2,75681031 | | Up |
| AFFX-PheX-3_at^a^ | 0,0000224 | *------* | | ------------------------------------------------ | | 2,75108364 | | Up |
| 1441816_at | 0,0000057 | *Kantr* | | Kdm5c adjacent non-coding transcript | | 2,73587061 | | Up |
| AFFX-PheX-M_at^a^ | 0,0000168 | *------* | | ------------------------------------------------ | | 2,68700685 | | Up |
| 1441445_at | 0,000021 | *Per3* | | Period circadian clock 3 | | 2,46912521 | | Up |
| 1455812_x_at | 0,00000212 | *Vasn* | | Vasorin | | 2,26262893 | | Up |
| 1437274_at | 0,00000436 | *Copa* | | Coatomer protein complex subunit alpha | | 2,2376742 | | Up |
| 1441021_at | 0,0000271 | *Letmd1* | | LETM1 domain containing 1 | | 2,21146131 | | Up |
| 1434512_x_at | 0,0000174 | *Srsf3* | | Serine/arginine-rich splicing factor 3 | | 2,10672207 | | Up |
| 1429680_at | 0,00000455 | *Tra2a* | | Transformer 2 alpha homolog (*Drosophila*) | | 2,10234582 | | Up |
| 1419719_at | 0,00000398 | *Gabrb1* | | Gamma-aminobutyric acid (GABA) A receptor, subunit beta 1 | | 2,04485706 | | Up |
| 1436698_x_at | 0,0000222 | *Tmem204* | | Transmembrane protein 204 | | 2,0265138 | | Up |
| 1434452_x_at | 0,000023 | *Eif2a* | | Eukaryotic translation initiation factor 2A | | 1,98068574 | | Up |
| 1438511_a_at | 0,00000113 | *Rgcc* | | Regulator of cell cycle | | 1,97109867 | | Up |
| 1441233_at | 0,0000242 | *Gm19858* | | Predicted gene, 19858 | | 1,97109867 | | Up |
| 1439090_at | 0,0000235 | *Tbc1d23* | | TBC1 domain family, member 23 | | 1,96427919 | | Up |
| 1422809_at | 0,00000253 | *Rims2* | | Regulating synaptic membrane exocytosis 2 | | 1,86735989 | | Up |
| 1444615_x_at | 0,000016 | *Runx1t1* | | Runt-related transcription factor 1; translocated to, 1 (cyclin D-related) | | 1,86089932 | | Up |
| 1437850_a_at | 0,0000171 | *Cnbp* | | Cellular nucleic acid binding protein | | 1,85574695 | | Up |
| 1422542_at | 0,00000344 | *Gpr34* | | G protein-coupled receptor 34 | | 1,84292837 | | Up |
| 1455695_at | 0,00000965 | *St8sia1* | | ST8 alpha-N-acetyl-neuraminide alpha-2,8-sialyltransferase 1 | | 1,83527976 | | Up |
| 1455787_x_at | 0,0000145 | *Minpp1* | | Multiple inositol polyphosphate histidine phosphatase 1 | | 1,83527976 | | Up |
| 1447774_x_at | 0,0000084 | *Fam213a* | | Family with sequence similarity 213, member A | | 1,83400809 | | Up |
| 1428942_at | 0,00000818 | *Mt2* | | Metallothionein 2 | | 1,82513098 | | Up |
| 1452011_a_at | 0,0000187 | *Uxs1* | | UDP-glucuronate decarboxylase 1 | | 1,8188165 | | Up |
| 1455822_x_at | 0,0000163 | *Surf4* | | Surfeit gene 4 | | 1,81503831 | | Up |
| 1451991_at | 0,0000278 | *Epha7* | | Eph receptor A7 | | 1,81126797 | | Up |
| 1441948_x_at | 0,0000102 | *Zfand3* | | Zinc finger, AN1-type domain 3 | | 1,77645359 | | Up |
| 1437782_at | 0,0000219 | *Cntnap2* | | Contactin associated protein-like 2 | | 1,7666301 | | Up |
| 1458168_at | 0,00000612 | *Ufm1* | | Ubiquitin-fold modifier 1 | | 1,75929815 | | Up |
| 1416179_a_at | 0,0000197 | *Rdx* | | Radixin | | 1,7544271 | | Up |
| 1438945_x_at | 0,0000126 | *Gja1* | | Gap junction protein, alpha 1 | | 1,72189838 | | Up |
| 1441733_s_at | 0,0000269 | *Nup153* | | Nucleoporin 153 | | 1,71832151 | | Up |
| 1459981_s_at | 0,00000563 | *Rsbn1* | | Rosbin, round spermatid basic protein 1 | | 1,71356391 | | Up |
| 1435702_s_at | 0,0000107 | *Ywhae* | | Tyrosine 3-monooxygenase/tryptophan 5-monooxygenase activation protein, epsilon polypeptide | | 1,68763159 | | Up |
| 1436997_x_at | 0,0000198 | *Sh3bgrl* | | SH3-binding domain glutamic acid-rich protein like | | 1,67597427 | | Up |
| 1444726_at | 0,0000119 | *Krr1* | | KRR1, small subunit (SSU) processome component, homolog (yeast) | | 1,66555154 | | Up |
| 1416189_a_at | 0,0000265 | *Sec61a1* | | Sec61 alpha 1 subunit (*S. cerevisiae*) | | 1,66555154 | | Up |
| 1416151_at | 0,00000352 | *Srsf3* | | Serine/arginine-rich splicing factor 3 | | 1,66439747 | | Up |
| 1419912_s_at | 0,000026 | *Strap* | | Serine/threonine kinase receptor associated protein | | 1,65290064 | | Up |
| 1429477_at | 0,0000189 | *Ncaph2* | | Non-SMC condensin II complex, subunit H2 | | 1,6267584 | | Up |
| 1456244_x_at | 0,0000286 | *Glrx3* | | Glutaredoxin 3 | | 1,62337916 | | Up |
| 1451313_a_at | 0,0000184 | *Lgalsl* | | Lectin, galactoside binding-like | | 1,62225431 | | Up |
| 1437992_x_at | 0,0000158 | *Gja1* | | Gap junction protein, alpha 1 | | 1,61328352 | | Up |
| 1418887_a_at | 0,0000173 | *Fam104a* | | Family with sequence similarity 104, member A | | 1,61104858 | | Up |
| 1437163_x_at | 0,000022 | *Gtf2h4* | | General transcription factor II H, polypeptide 4 | | 1,59881166 | | Up |
| 1452209_at | 0,0000242 | *Pkp4* | | Plakophilin 4 | | 1,57898777 | | Up |
| 1438553_x_at | 0,000026 | *4930453N24Rik* | | RIKEN cDNA 4930453N24 gene | | 1,57461595 | | Up |
| 1438984_x_at | 0,0000264 | *Psmb4* | | Proteasome (prosome, macropain) subunit, beta type 4 | | 1,55078641 | | Up |
| 1423696_a_at | 0,0000283 | *Psmd6* | | Proteasome (prosome, macropain) 26S subunit, non-ATPase, 6 | | 1,54649267 | | Up |
| 1436848_x_at | 0,0000255 | *Impa1* | | Inositol (myo)-1(or 4)-monophosphatase 1 | | 1,54435027 | | Up |
| 1448210_at | 0,0000119 | *Rab1* | | RAB1, member RAS oncogene family | | 1,51781925 | | Up |
| 1459854_s_at | 0,0000218 | *Dynlt3* | | Dynein light chain Tctex-type 3 | | 1,50524675 | | Up |
| 1433796_at | 0,0000275 | *Endod1* | | Endonuclease domain containing 1 | | 1,49588576 | | Up |
| 1438637_x_at | 0,0000134 | *Sf3b2* | | Splicing factor 3b, subunit 2 | | 1,49174403 | | Up |
| 1425116_a_at | 0,0000244 | *Sptbn4* | | Spectrin beta, non-erythrocytic 4 | | 0,64483413 | | Down |
| 1424180_a_at | 0,000017 | *Med24* | | Mediator complex subunit 24 | | 0,63683874 | | Down |
| 1452100_at | 0,0000263 | *Ctdnep1* | | CTD nuclear envelope phosphatase 1 | | 0,63243977 | | Down |
| 1417274_at | 0,00000799 | *Snrpa* | | Small nuclear ribonucleoprotein polypeptide A | | 0,6306887 | | Down |
| 1416517_at | 0,0000126 | *Pnpla6* | | Patatin-like phospholipase domain containing 6 | | 0,62850669 | | Down |
| 1422521_at | 0,000023 | *Dctn1* | | Dynactin 1 | | 0,6272011 | | Down |
| 1434643_at | 0,0000224 | *Tbl1x* | | Transducin (beta)-like 1 X-linked | | 0,62416527 | | Down |
| 1434154_at | 0,00000957 | *Kctd13* | | Potassium channel tetramerisation domain containing 13 | | 0,61770932 | | Down |
| 1416900_s_at | 0,00000958 | *Cers1///Gdf1* | | Ceramide synthase 1///growth differentiation factor 1 | | 0,61770932 | | Down |
| 1423983_at | 0,0000134 | *Sez6l2* | | Seizure related 6 homolog like 2 | | 0,61642616 | | Down |
| 1419866_s_at | 0,0000282 | *Atxn2* | | Ataxin 2 | | 0,61429349 | | Down |
| 1417423_at | 0,00000358 | *Grina* | | Glutamate receptor, ionotropic, N-methyl D-aspartate-associated protein 1 (glutamate binding) | | 0,60878301 | | Down |
| 1428147_at | 0,00000379 | *Coro7* | | Coronin 7 | | 0,60332196 | | Down |
| 1452375_at | 0,0000266 | *Aldh4a1* | | Aldehyde dehydrogenase 4 family, member A1 | | 0,60248616 | | Down |
| 1451291_at | 0,0000079 | *Nabp2* | | Nucleic acid binding protein 2 | | 0,60123462 | | Down |
| 1438022_at | 0,0000223 | *Rab11fip3* | | RAB11 family interacting protein 3 (class II) | | 0,5974956 | | Down |
| 1415956_a_at | 0,0000123 | *Cdk16* | | Cyclin-dependent kinase 16 | | 0,59542842 | | Down |
| 1424622_at | 0,00000804 | *Hsf1* | | Heat shock factor 1 | | 0,59501585 | | Down |
| 1417295_at | 0,0000241 | *Mta1* | | Metastasis associated 1 | | 0,59419155 | | Down |
| 1417709_at | 0,00000837 | *Cyp46a1* | | Cytochrome P450, family 46, subfamily a, polypeptide 1 | | 0,5933684 | | Down |
| 1421164_a_at | 0,0000244 | *Arhgef1* | | Rho guanine nucleotide exchange factor (GEF) 1 | | 0,59213581 | | Down |
| 1456909_at | 0,00000858 | *Gpi1* | | Glucose phosphate isomerase 1 | | 0,58886139 | | Down |
| 1449290_at | 0,0000272 | *Dpysl5* | | Dihydropyrimidinase-like 5 | | 0,58845337 | | Down |
| 1451592_at | 0,0000181 | *Mypop* | | Myb-related transcription factor, partner of profilin | | 0,58519932 | | Down |
| 1424040_at | 0,0000137 | *Map7d1* | | MAP7 domain containing 1 | | 0,5839837 | | Down |
| 1450519_a_at | 0,0000154 | *Prkaca* | | Protein kinase, cAMP dependent, catalytic, alpha | | 0,5839837 | | Down |
| 1423087_a_at | 0,000015 | *Prickle4///Tomm6* | | Prickle homolog 4 (Drosophila)///translocase of outer mitochondrial membrane 6 homolog (yeast) | | 0,5791464 | | Down |
| 1427103_at | 0,00000865 | *Plekho2* | | Pleckstrin homology domain containing, family O member 2 | | 0,57874511 | | Down |
| 1431285_at | 0,00000539 | *Mgrn1* | | Mahogunin, ring finger 1 | | 0,57834409 | | Down |
| 1451841_a_at | 0,0000204 | *Ncor2* | | Nuclear receptor co-repressor 2 | | 0,57634317 | | Down |
| 1448280_at | 0,00000568 | *Syp* | | Synaptophysin | | 0,57594382 | | Down |
| 1449362_a_at | 0,00000296 | *Mink1* | | Misshapen-like kinase 1 (zebrafish) | | 0,57395121 | | Down |
| 1452058_a_at | 0,0000173 | *Rnf11* | | Ring finger protein 11 | | 0,57275895 | | Down |
| 1419213_at | 0,00000969 | *Nat6* | | N-acetyltransferase 6 | | 0,57236208 | | Down |
| 1449272_at | 0,000026 | *Cadm3* | | Cell adhesion molecule 3 | | 0,57117312 | | Down |
| 1417747_at | 0,0000181 | *Cplx1* | | Complexin 1 | | 0,57038186 | | Down |
| 1438058_s_at | 0,00000982 | *Ptov1* | | Prostate tumor over expressed gene 1 | | 0,56762105 | | Down |
| 1418284_at | 0,0000184 | *Vps72* | | Vacuolar protein sorting 72 (yeast) | | 0,56526528 | | Down |
| 1416365_at | 0,0000146 | *Hsp90ab1* | | Heat shock protein 90 alpha (cytosolic), class B member 1 | | 0,56487361 | | Down |
| 1455657_at | 0,00000675 | *Smg1* | | SMG1 homolog, phosphatidylinositol 3-kinase-related kinase (*C. elegans*) | | 0,56291929 | | Down |
| 1434992_at | 0,0000108 | *Zfr2* | | Zinc finger RNA binding protein 2 | | 0,56252924 | | Down |
| 1426329_s_at | 0,0000188 | *Baalc* | | Brain and acute leukemia, cytoplasmic | | 0,56097174 | | Down |
| 1426184_a_at | 0,000024 | *Pdcd6ip* | | Programmed cell death 6 interacting protein | | 0,55748311 | | Down |
| 1436059_at | 0,0000127 | *Rfx1* | | Regulatory factor X, 1 (influences HLA class II expression) | | 0,55478474 | | Down |
| 1421312_a_at | 0,0000143 | *Kifc2* | | Kinesin family member C2 | | 0,55440032 | | Down |
| 1424619_at | 0,0000233 | *Sf3b4* | | Splicing factor 3b, subunit 4 | | 0,55440032 | | Down |
| 1427079_at | 0,0000242 | *Mapre3* | | Microtubule-associated protein, RP/EB family, member 3 | | 0,55401617 | | Down |
| 1448121_at | 0,0000218 | *Wbp2* | | WW domain binding protein 2 | | 0,55209942 | | Down |
| 1460169_a_at | 0,0000024 | *Cdk16* | | Cyclin-dependent kinase 16 | | 0,54980807 | | Down |
| 1455714_at | 0,00000333 | *Vstm2l* | | V-set and transmembrane domain containing 2-like | | 0,54866597 | | Down |
| 1450451_at | 0,0000104 | *Spock2* | | Sparc/osteonectin, cwcv and kazal-like domains proteoglycan 2 | | 0,54790588 | | Down |
| 1451476_at | 0,00000756 | *Zdhhc8* | | Zinc finger, DHHC domain containing 8 | | 0,54752624 | | Down |
| 1449054_a_at | 0,00000934 | *Pcbp4* | | Poly(rC) binding protein 4 | | 0,54714685 | | Down |
| 1435784_at | 0,00000984 | *Atg9a* | | Autophagy related 9A | | 0,54676773 | | Down |
| 1428707_at | 0,0000145 | *Ptms* | | Parathymosin | | 0,54563194 | | Down |
| 1415757_at | 0,0000108 | *Gbf1* | | Golgi-specific brefeldin A-resistance factor 1 | | 0,54525387 | | Down |
| 1427988_s_at | 0,00000363 | *Safb2* | | Scaffold attachment factor B2 | | 0,5437442 | | Down |
| 1416134_at | 0,0000227 | *Aplp1* | | Amyloid beta (A4) precursor-like protein 1 | | 0,54261469 | | Down |
| 1452921_at | 0,00000478 | *LOC100503956///Evi5l* | | EVI5-like protein-like///ecotropic viral integration site 5 like | | 0,54186298 | | Down |
| 1425760_a_at | 0,0000235 | *Pitpnm1* | | Phosphatidylinositol transfer protein, membrane-associated 1 | | 0,54148752 | | Down |
| 1449511_a_at | 0,00000516 | *Ssbp4* | | Single stranded DNA binding protein 4 | | 0,53998828 | | Down |
| 1439583_x_at | 0,00000657 | *Rltpr* | | RGD motif, leucine rich repeats, tropomodulin domain and proline-rich containing | | 0,53924022 | | Down |
| 1424490_at | 0,00000215 | *Zfp428* | | Zinc finger protein 428 | | 0,53886657 | | Down |
| 1450903_at | 0,0000145 | *Rad23b* | | RAD23b homolog (*S. cerevisiae*) | | 0,53849319 | | Down |
| 1426807_at | 0,0000188 | *Lta4h* | | Leukotriene A4 hydrolase | | 0,53812006 | | Down |
| 1426122_a_at | 0,0000109 | *Coro6* | | Coronin 6 | | 0,53366267 | | Down |
| 1451168_a_at | 0,0000114 | *Arhgdia* | | Rho GDP dissociation inhibitor (GDI) alpha | | 0,53144784 | | Down |
| 1428513_at | 0,0000117 | *Calcoco1* | | Calcium binding and coiled coil domain 1 | | 0,52960917 | | Down |
| 1421154_at | 0,0000181 | *Hcn2* | | Hyperpolarization-activated, cyclic nucleotide-gated K+ 2 | | 0,5292422 | | Down |
| 1450202_at | 6,68E-07 | *Grin1* | | Glutamate receptor, ionotropic, NMDA1 (zeta 1) | | 0,52814281 | | Down |
| 1424203_at | 0,0000023 | *Ncln* | | Nicalin homolog (zebrafish) | | 0,52741116 | | Down |
| 1423630_at | 0,0000247 | *Cygb* | | Cytoglobin | | 0,52631558 | | Down |
| 1426843_at | 0,0000146 | *Atg2a* | | Autophagy related 2A | | 0,52485834 | | Down |
| 1426024_a_at | 0,00000511 | *Dbn1* | | Drebrin 1 | | 0,52231788 | | Down |
| 1425679_a_at | 0,00000362 | *Mapk8ip1* | | Mitogen-activated protein kinase 8 interacting protein 1 | | 0,51978972 | | Down |
| 1434610_at | 0,0000224 | *Plec* | | Plectin | | 0,51978972 | | Down |
| 1444046_at | 0,0000132 | *Shisa7* | | Shisa homolog 7 (*Xenopus laevis*) | | 0,51870997 | | Down |
| 1416515_at | 0,0000184 | *Fscn1* | | Fascin homolog 1, actin bundling protein (*Strongylocentrotus purpuratus*) | | 0,51655719 | | Down |
| 1452235_at | 0,00000281 | *Man1b1* | | Mannosidase, alpha, class 1B, member 1 | | 0,51548416 | | Down |
| 1416965_at | 0,00000612 | *Pcsk1n* | | Proprotein convertase subtilisin/kexin type 1 inhibitor | | 0,51405691 | | Down |
| 1423559_at | 0,000019 | *Kcnc1* | | Potassium voltage gated channel, Shaw-related subfamily, member 1 | | 0,51405691 | | Down |
| 1427338_at | 0,00000801 | *Crocc* | | Ciliary rootlet coiled-coil, rootletin | | 0,51227841 | | Down |
| 1419651_at | 0,0000184 | *Nkain1* | | Na+/K+ transporting ATPase interacting 1 | | 0,51192345 | | Down |
| 1421149_a_at | 0,0000279 | *Rnu7///Atn1* | | U7 small nuclear RNA///atrophin 1 | | 0,51156873 | | Down |
| 1419580_at | 0,00000781 | *Dlg4* | | Discs, large homolog 4 (*Drosophila*) | | 0,51015233 | | Down |
| 1460210_at | 0,00000527 | *Pkd1* | | Polycystic kidney disease 1 homolog | | 0,5090926 | | Down |
| 1427902_at | 0,00000176 | *Srrm2* | | Serine/arginine repetitive matrix 2 | | 0,50873985 | | Down |
| 1436205_at | 0,00000853 | *Nfasc* | | Neurofascin | | 0,50662845 | | Down |
| 1428708_x_at | 0,0000204 | *Ptms* | | Parathymosin | | 0,50522572 | | Down |
| 1425777_at | 0,00000118 | *Cacnb1* | | Calcium channel, voltage-dependent, beta 1 subunit | | 0,50452582 | | Down |
| 1416437_a_at | 0,0000101 | *Mapk8ip3* | | Mitogen-activated protein kinase 8 interacting protein 3 | | 0,50138822 | | Down |
| 1438668_x_at | 3,37E-07 | *Atxn2l* | | Ataxin 2-like | | 0,49792488 | | Down |
| 1448941_at | 0,00000723 | *B4galt2* | | UDP-Gal:betaGlcNAc beta 1,4- galactosyltransferase, polypeptide 2 | | 0,49585837 | | Down |
| 1440407_at^a^ | 0,00000452 | *------* | | ------------------------------------------------ | | 0,49517144 | | Down |
| 1417632_at | 0,00000558 | *Atp6v0a1* | | ATPase, H+ transporting, lysosomal V0 subunit A1 | | 0,49380043 | | Down |
| 1420619_a_at | 0,00000169 | *Aes* | | Amino-terminal enhancer of split | | 0,49345827 | | Down |
| 1423530_at | 0,00000193 | *Stk32c* | | Serine/threonine kinase 32C | | 0,48869288 | | Down |
| 1424640_at | 0,00000208 | *Arl8a* | | ADP-ribosylation factor-like 8A | | 0,48801588 | | Down |
| 1423221_at | 0,0000249 | *Tubb4a* | | Tubulin, beta 4A class IVA | | 0,48801588 | | Down |
| 1455942_at | 0,0000101 | *Kdm2a* | | Lysine (K)-specific demethylase 2A | | 0,48666469 | | Down |
| 1434032_at | 0,00000122 | *Agap3* | | ArfGAP with GTPase domain, ankyrin repeat and PH domain 3 | | 0,48531723 | | Down |
| 1431092_at | 0,0000115 | *Ppp1r12c* | | Protein phosphatase 1, regulatory (inhibitor) subunit 12C | | 0,48397351 | | Down |
| 1419742_at | 0,000011 | *1700037H04Rik* | | RIKEN cDNA 1700037H04 gene | | 0,48296816 | | Down |
| 1419743_s_at | 0,0000165 | *Carm1* | | Coactivator-associated arginine methyltransferase 1 | | 0,48129722 | | Down |
| 1426873_s_at | 0,00000392 | *Jup* | | Junction plakoglobin | | 0,47929972 | | Down |
| 1424666_at | 0,0000115 | *Gpatch8* | | G patch domain containing 8 | | 0,47863573 | | Down |
| 1427265_at | 0,000015 | *Bcr* | | Breakpoint cluster region | | 0,47797266 | | Down |
| 1452827_at | 4,92E-07 | *1500009C09Rik* | | RIKEN cDNA 1500009C09 gene | | 0,476319 | | Down |
| 1422635_at | 0,00000322 | *Ache* | | Acetylcholinesterase | | 0,476319 | | Down |
| 1456800_a_at | 0,00000788 | *Rltpr* | | RGD motif, leucine rich repeats, tropomodulin domain and proline-rich containing | | 0,47401348 | | Down |
| 1426493_a_at | 6,36E-07 | *Kifc2* | | Kinesin family member C2 | | 0,47302882 | | Down |
| 1454206_a_at | 0,00000802 | *Adam15* | | Disintegrin and metallopeptidase domain 15 (metargidin) | | 0,47302882 | | Down |
| 1460262_a_at | 0,00000275 | *1700037H04Rik* | | RIKEN cDNA 1700037H04 gene | | 0,47270106 | | Down |
| 1434531_at | 0,00000586 | *Mgat5b* | | Mannoside acetylglucosaminyltransferase 5, isoenzyme B | | 0,47139227 | | Down |
| 1430543_at | 7,52E-07 | *Clip3* | | CAP-GLY domain containing linker protein 3 | | 0,47041305 | | Down |
| 1426617_a_at | 8,83E-07 | *Ttyh1* | | Tweety homolog 1 (*Drosophila*) | | 0,46943587 | | Down |
| 1418078_at | 0,00000644 | *Psme3* | | Proteaseome (prosome, macropain) activator subunit 3 (PA28 gamma, Ki) | | 0,46878555 | | Down |
| 1430649_at | 0,00000774 | *Baalc* | | Brain and acute leukemia, cytoplasmic | | 0,46813612 | | Down |
| 1449030_at | 0,00000207 | *Syn2* | | Synapsin II | | 0,46393674 | | Down |
| 1455463_at | 0,000001 | *Phyhip* | | Phytanoyl-CoA hydroxylase interacting protein | | 0,46361527 | | Down |
| 1427039_at | 0,00000449 | *Epn1* | | Epsin 1 | | 0,46361527 | | Down |
| 1425711_a_at | 0,00000972 | *Akt1* | | Thymoma viral proto-oncogene 1 | | 0,46137125 | | Down |
| 1451872_a_at | 0,0000183 | *Neurl1a* | | Neuralized homolog 1A (*Drosophila*) | | 0,45881994 | | Down |
| 1426791_at | 0,0000229 | *Rusc2* | | RUN and SH3 domain containing 2 | | 0,45850202 | | Down |
| 1422564_at | 0,00000181 | *Actl6b* | | Actin-like 6B | | 0,45723254 | | Down |
| 1458443_at | 0,00000708 | *Crtc1* | | CREB regulated transcription coactivator 1 | | 0,45596658 | | Down |
| 1437968_at | 0,00000329 | *Grin1* | | Glutamate receptor, ionotropic, NMDA1 (zeta 1) | | 0,45565064 | | Down |
| 1418015_at | 0,00000304 | *Pum2* | | Pumilio RNA-binding family member 2 | | 0,45407421 | | Down |
| 1451992_at | 0,000014 | *Adrbk1* | | Adrenergic receptor kinase, beta 1 | | 0,45093769 | | Down |
| 1443170_at | 0,000011 | *Cnnm1* | | Cyclin M1 | | 0,44968916 | | Down |
| 1418356_at | 0,00000402 | *Mpst* | | Mercaptopyruvate sulfurtransferase | | 0,44782284 | | Down |
| 1459003_at | 0,0000184 | *Fhl1* | | Four and a half LIM domains 1 | | 0,44534645 | | Down |
| 1436077_a_at | 0,00000534 | *Fcho1* | | FCH domain only 1 | | 0,44503787 | | Down |
| 1439748_at | 9,62E-07 | *Dpp6* | | Dipeptidylpeptidase 6 | | 0,44196377 | | Down |
| 1460724_at | 6,22E-07 | *Ap2a1* | | Adaptor-related protein complex 2, alpha 1 subunit | | 0,43951978 | | Down |
| 1422034_a_at | 7,33E-07 | *Palm* | | Paralemmin | | 0,43830286 | | Down |
| 1422109_at | 0,0000192 | *Rfx1* | | Regulatory factor X, 1 (influences HLA class II expression) | | 0,43708931 | | Down |
| 1455363_at | 0,0000197 | *Bai1* | | Brain-specific angiogenesis inhibitor 1 | | 0,43618135 | | Down |
| 1420924_at | 0,00000166 | *Timp2* | | Tissue inhibitor of metalloproteinase 2 | | 0,4355771 | | Down |
| 1460650_at | 0,0000155 | *Atp6v0a1* | | ATPase, H+ transporting, lysosomal V0 subunit A1 | | 0,43017632 | | Down |
| 1436084_at | 0,0000053 | *Scrt1* | | Scratch homolog 1, zinc finger protein (*Drosophila*) | | 0,42898527 | | Down |
| 1435026_at | 0,00000664 | *Spock2* | | Sparc/osteonectin, cwcv and kazal-like domains proteoglycan 2 | | 0,42750109 | | Down |
| 1421915_a_at | 0,000025 | *St3gal3* | | ST3 beta-galactoside alpha-2,3-sialyltransferase 3 | | 0,42543186 | | Down |
| 1417524_at | 2,27E-08 | *Cnih2* | | Cornichon homolog 2 (*Drosophila*) | | 0,42513708 | | Down |
| 1456304_at | 2,38E-07 | *Gm996* | | Predicted gene 996 | | 0,42278614 | | Down |
| 1452415_at | 0,00000203 | *Actn1* | | Actinin, alpha 1 | | 0,42220044 | | Down |
| 1418214_at | 0,00000745 | *Klc2* | | Kinesin light chain 2 | | 0,41870324 | | Down |
| 1431137_at | 0,00000806 | *Rusc1* | | RUN and SH3 domain containing 1 | | 0,41870324 | | Down |
| 1421789_s_at | 0,0000204 | *Arf3* | | ADP-ribosylation factor 3 | | 0,41841312 | | Down |
| 1451465_at | 0,000006 | *Ubl7* | | Ubiquitin-like 7 (bone marrow stromal cell-derived) | | 0,41696552 | | Down |
| 1437491_at | 0,00000505 | *Bicd2* | | Bicaudal D homolog 2 (*Drosophila*) | | 0,4103708 | | Down |
| 1429634_at | 0,00000197 | *Zfp580* | | Zinc finger protein 580 | | 0,40781875 | | Down |
| 1421160_a_at | 0,00000181 | *Rfng* | | RFNG O-fucosylpeptide 3-beta-N-acetylglucosaminyltransferase | | 0,40388039 | | Down |
| 1441312_at | 8,45E-07 | *Cnnm1* | | Cyclin M1 | | 0,401647 | | Down |
| 1436665_a_at | 0,0000232 | *Ltbp4* | | Latent transforming growth factor beta binding protein 4 | | 0,40136869 | | Down |
| 1453527_a_at | 0,00000357 | *Neurl1a* | | Neuralized homolog 1A (*Drosophila*) | | 0,39998006 | | Down |
| 1451795_at | 0,0000247 | *Tom1l2* | | Target of myb1-like 2 (Chicken) | | 0,39942596 | | Down |
| 1429719_at | 0,0000173 | *Foxp4* | | Forkhead box P4 | | 0,39832005 | | Down |
| 1419581_at | 0,00000163 | *Dlg4* | | Discs, large homolog 4 (*Drosophila*) | | 0,39338122 | | Down |
| 1422799_at | 0,00000141 | *Prrc2a* | | Proline-rich coiled-coil 2A | | 0,39202023 | | Down |
| 1425337_at | 0,00000534 | *Slc12a5* | | Solute carrier family 12, member 5 | | 0,39174859 | | Down |
| 1421267_a_at | 0,00000148 | *Cited2* | | Cbp/p300-interacting transactivator, with Glu/Asp-rich carboxy-terminal domain, 2 | | 0,38958229 | | Down |
| 1425690_at | 0,00000394 | *B3gat1* | | Beta-1,3-glucuronyltransferase 1 (glucuronosyltransferase P) | | 0,38958229 | | Down |
| 1434908_at | 0,00000951 | *Scaf1* | | SR-related CTD-associated factor 1 | | 0,38877302 | | Down |
| 1422647_at | 0,00000496 | *Ring1* | | Ring finger protein 1 | | 0,38689125 | | Down |
| 1436780_at | 0,00000176 | *Ogt* | | O-linked N-acetylglucosamine (GlcNAc) transferase (UDP-N-acetylglucosamine:polypeptide-N-acetylglucosaminyl transferase) | | 0,37735649 | | Down |
| 1422073_a_at | 0,00000352 | *Celsr2* | | Cadherin, EGF LAG seven-pass G-type receptor 2 (flamingo homolog, *Drosophila*) | | 0,37605094 | | Down |
| 1425724_at | 0,00000138 | *Ptprn2* | | Protein tyrosine phosphatase, receptor type, N polypeptide 2 | | 0,37500975 | | Down |
| 1425276_at | 0,00000181 | *Fbrs* | | Fibrosin | | 0,3747499 | | Down |
| 1423561_at | 0,0000154 | *Nell2* | | NEL-like 2 | | 0,37164575 | | Down |
| 1425535_at | 0,00000239 | *Repin1* | | Replication initiator 1 | | 0,3690786 | | Down |
| 1426336_at | 0,0000123 | *Cacng7* | | Calcium channel, voltage-dependent, gamma subunit 7 | | 0,365008 | | Down |
| 1427688_a_at | 0,00000414 | *Ptprs* | | Protein tyrosine phosphatase, receptor type, S | | 0,35898613 | | Down |
| 1422321_a_at | 0,00000168 | *Sf1* | | Splicing factor 1 | | 0,35799219 | | Down |
| 1417963_at | 0,00000401 | *Pltp* | | Phospholipid transfer protein | | 0,34460129 | | Down |
| 1422009_at | 2,72E-07 | *Atp1b2* | | ATPase, Na+/K+ transporting, beta 2 polypeptide | | 0,34293332 | | Down |
| 1420833_at | 1,21E-07 | *Vamp2* | | Vesicle-associated membrane protein 2 | | 0,33564313 | | Down |
| 1435152_at | 0,0000108 | *Leng8* | | Leukocyte receptor cluster (LRC) member 8 | | 0,33448189 | | Down |
| 1421368_at | 0,0000098 | *Scrt1* | | Scratch homolog 1, zinc finger protein (*Drosophila*) | | 0,33194129 | | Down |
| 1455499_at | 0,0000212 | *Nrxn2* | | Neurexin II | | 0,32987698 | | Down |
| 1427099_at | 0,0000176 | *Maz* | | MYC-associated zinc finger protein (purine-binding transcription factor) | | 0,32714452 | | Down |
| 1449173_at | 0,00000226 | *Mpp2* | | Membrane protein, palmitoylated 2 (MAGUK p55 subfamily member 2) | | 0,32152437 | | Down |
| 1427754_a_at | 0,00000079 | *Dnm1* | | Dynamin 1 | | 0,30481377 | | Down |
| 1451734_a_at | 5,65E-07 | *Dbn1* | | Drebrin 1 | | 0,29647862 | | Down |
| 1420575_at | 0,00000483 | *Mt3* | | Metallothionein 3 | | 0,29199185 | | Down |
| 1442066_at | 0,00000315 | *Pianp* | | PILR alpha associated neural protein | | 0,2718721 | | Down |
| 1426538_a_at | 0,000019 | *Trp53* | | Transformation related protein 53 | | 0,2582786 | | Down |
| 1420964_at | 4,07E-07 | *Enc1* | | Ectodermal-neural cortex 1 | | 0,23116582 | | Down |
| 1427481_a_at | 6,25E-07 | *Atp1a3* | | ATPase, Na+/K+ transporting, alpha 3 polypeptide | | 0,21345443 | | Down |
| 1433308_at | 0,00000321 | *9330159N22Rik* | | RIKEN cDNA 9330159N22 gene | | 0,14958112 | | Down |
| 1428054_at | 0,0000114 | *Slc8a2* | | Solute carrier family 8 (sodium/calcium exchanger), member 2 | | 0,11809338 | | Down |
| 1430197_a_at | 0,0000244 | *Pitpnm2* | | Phosphatidylinositol transfer protein, membrane-associated 2 | | 0,11250024 | | Down |
| 1439051_a_at | 4,61E-07 | *Mark4* | | MAP/microtubule affinity-regulating kinase 4 | | 0,06232695 | | Down |

* Resveratrol-enriched *versus* control diet

**^a^** Neither symbol nor title assigned to this probe
