# Supplementary material for: A genomics approach identifies selective effects of trans-resveratrol in cerebral cortex neuron and glia gene expression
Source: PLoS One. 2017 Apr 25;12(4):e0176067. doi: 10.1371/journal.pone.0176067 (PMC5404873; doi:10.1371/journal.pone.0176067)
Supplement: S1 Text — (DOCX) [file pone.0176067.s003.docx]

**S1 Text. Data analysis for S2 Table.**

S2 Table shows raw results of 250 differentially expressed genes ranked by p-value. The table includes the following parameters:

- p-value after adjustment for multiple testing. Pairwise comparisons (with/without RSV) providing the smallest p-values (the smaller and the most reliable) were computed.

- Moderated t-statistic. This test is similar to the Student’s t-test as it compares the gene expression mean values for two individual replicates or two groups of replicates for a given gene. The primary difference between the two methods is in the calculation of variance. The Student’s t-test calculates variance from the data that is available for each gene. The Moderated t-test uses information from all of the selected genes to calculate variance. The moderated t-statistic has been extremely useful in analyzing data from microarray experiments.

- logFC: Base 2 log of the fold change (FC) of gene expression in the two experimental conditions (with/without RSV).
