# Supplementary material for: A genomics approach identifies selective effects of trans-resveratrol in cerebral cortex neuron and glia gene expression
Source: PLoS One. 2017 Apr 25;12(4):e0176067. doi: 10.1371/journal.pone.0176067 (PMC5404873; doi:10.1371/journal.pone.0176067)
Supplement: S2 Text — (DOCX) [file pone.0176067.s004.docx]

**S2 Text. R script provided by GEO2R analysis.**

# Version info: R 3.2.3, Biobase 2.30.0, GEOquery 2.40.0, limma 3.26.8

# R scripts generated Wed Mar 8 14:03:50 EST 2017

################################################################

# Differential expression analysis with limma

library(Biobase)

library(GEOquery)

library(limma)

# load series and platform data from GEO

gset <- getGEO("GSE11291", GSEMatrix =TRUE, AnnotGPL=TRUE)

if (length(gset) > 1) idx <- grep("GPL1261", attr(gset, "names")) else idx <- 1

gset <- gset[[idx]]

# make proper column names to match toptable

fvarLabels(gset) <- make.names(fvarLabels(gset))

# group names for all samples

gsms <- "XXXXXXXXXXXXXXXXXXXXXXXXXXXXXXXXXXXXXXXXXXXXX00000XXXXX11111"

sml <- c()

for (i in 1:nchar(gsms)) { sml[i] <- substr(gsms,i,i) }

# eliminate samples marked as "X"

sel <- which(sml != "X")

sml <- sml[sel]

gset <- gset[ ,sel]

# log2 transform

ex <- exprs(gset)

qx <- as.numeric(quantile(ex, c(0., 0.25, 0.5, 0.75, 0.99, 1.0), na.rm=T))

LogC <- (qx[5] > 100) ||

(qx[6]-qx[1] > 50 && qx[2] > 0) ||

(qx[2] > 0 && qx[2] < 1 && qx[4] > 1 && qx[4] < 2)

if (LogC) { ex[which(ex <= 0)] <- NaN

exprs(gset) <- log2(ex) }

# set up the data and proceed with analysis

sml <- paste("G", sml, sep="") # set group names

fl <- as.factor(sml)

gset$description <- fl

design <- model.matrix(~ description + 0, gset)

colnames(design) <- levels(fl)

fit <- lmFit(gset, design)

cont.matrix <- makeContrasts(G1-G0, levels=design)

fit2 <- contrasts.fit(fit, cont.matrix)

fit2 <- eBayes(fit2, 0.01)

tT <- topTable(fit2, adjust="fdr", sort.by="B", number=250)

tT <- subset(tT, select=c("ID","adj.P.Val","P.Value","t","B","logFC","Gene.symbol","Gene.title"))

write.table(tT, file=stdout(), row.names=F, sep="\t")

################################################################

# Boxplot for selected GEO samples

library(Biobase)

library(GEOquery)

# load series and platform data from GEO

gset <- getGEO("GSE11291", GSEMatrix =TRUE, getGPL=FALSE)

if (length(gset) > 1) idx <- grep("GPL1261", attr(gset, "names")) else idx <- 1

gset <- gset[[idx]]

# group names for all samples in a series

gsms <- "XXXXXXXXXXXXXXXXXXXXXXXXXXXXXXXXXXXXXXXXXXXXX00000XXXXX11111"

sml <- c()

for (i in 1:nchar(gsms)) { sml[i] <- substr(gsms,i,i) }

sml <- paste("G", sml, sep="") set group names

# eliminate samples marked as "X"

sel <- which(sml != "X")

sml <- sml[sel]

gset <- gset[ ,sel]

# order samples by group

ex <- exprs(gset)[ , order(sml)]

sml <- sml[order(sml)]

fl <- as.factor(sml)

labels <- c("control","rsv")

# set parameters and draw the plot

palette(c("#dfeaf4","#f4dfdf", "#AABBCC"))

dev.new(width=4+dim(gset)[[2]]/5, height=6)

par(mar=c(2+round(max(nchar(sampleNames(gset)))/2),4,2,1))

title <- paste ("GSE11291", '/', annotation(gset), " selected samples", sep ='')

boxplot(ex, boxwex=0.6, notch=T, main=title, outline=FALSE, las=2, col=fl)

legend("topleft", labels, fill=palette(), bty="n")
