# Supplementary material for: A genomics approach identifies selective effects of trans-resveratrol in cerebral cortex neuron and glia gene expression
Source: PLoS One. 2017 Apr 25;12(4):e0176067. doi: 10.1371/journal.pone.0176067 (PMC5404873; doi:10.1371/journal.pone.0176067)

**S1 Fig. Network analysis using STRING and the non-curated list of differentially expressed genes in neocortex after RSV diet.**


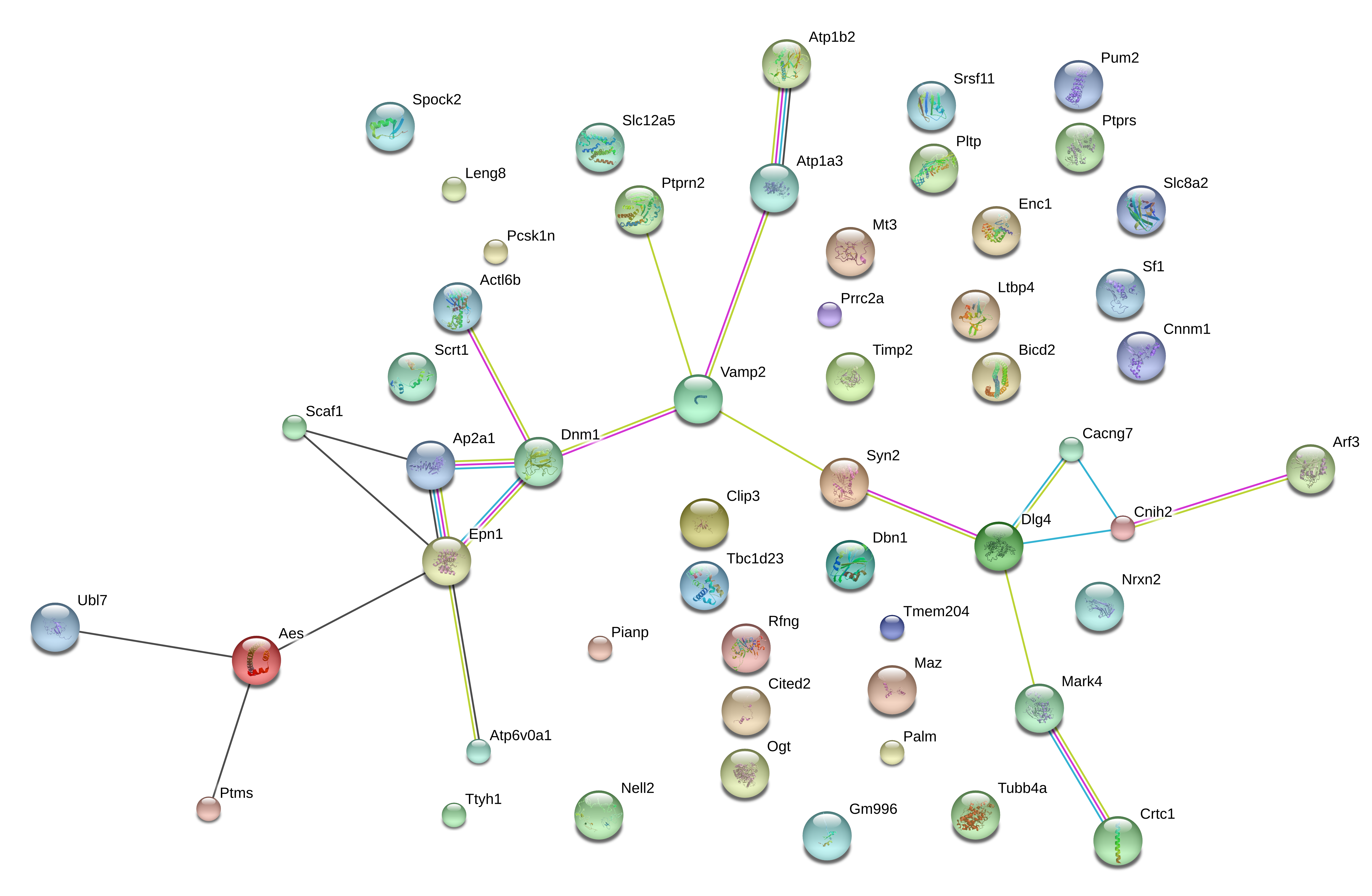

Supplement: S1 Fig — (DOCX) [file pone.0176067.s005.docx]
